# Seeing the wood despite the trees: Exploring human disturbance impact on plant diversity, community structure, and standing biomass in fragmented high Andean forests

**DOI:** 10.1002/ece3.7182

**Published:** 2021-02-01

**Authors:** Mariasole Calbi, Francisco Fajardo‐Gutiérrez, Juan Manuel Posada, Robert Lücking, Grischa Brokamp, Thomas Borsch

**Affiliations:** ^1^ Botanischer Garten und Botanisches Museum Berlin Freie Universität Berlin Berlin Germany; ^2^ Institut für Biologie – Systematische Botanik und Pflanzengeographie Freie Universität Berlin Berlin Germany; ^3^ Jardín Botánico de Bogotá José Celestino Mutis Bogotá Colombia; ^4^ Biology Department Faculty of Natural Sciences Universidad del Rosario Bogotá Colombia

**Keywords:** aboveground biomass, biodiversity, *bosque altoandino*, Colombia, cryptic forest degradation, understory

## Abstract

High Andean forests harbor a remarkably high biodiversity and play a key role in providing vital ecosystem services for neighboring cities and settlements. However, they are among the most fragmented and threatened ecosystems in the neotropics. To preserve their unique biodiversity, a deeper understanding of the effects of anthropogenic perturbations on them is urgently needed. Here, we characterized the plant communities of high Andean forest remnants in the hinterland of Bogotá in 32 0.04 ha plots. We assessed the woody vegetation and sampled the understory and epiphytic cover. We gathered data on compositional and structural parameters and compiled a broad array of variables related to anthropogenic disturbance, ranging from local to landscape‐wide metrics. We also assessed phylogenetic diversity and functional diversity. We employed nonmetric multidimensional scaling (NMDS) to select meaningful variables in a first step of the analysis. Then, we performed partial redundancy analysis (pRDA) and generalized linear models (GLMs) in order to test how selected environmental and anthropogenic variables are affecting the composition, diversity, and aboveground biomass of these forests. Identified woody vegetation and understory layer communities were characterized by differences in elevation, temperature, and relative humidity, but were also related to different levels of human influence. We found that the increase of human‐related disturbance resulted in less phylogenetic diversity and in the phylogenetic clustering of the woody vegetation and in lower aboveground biomass (AGB) values. As to the understory, disturbance was associated with a higher diversity, jointly with a higher phylogenetic dispersion. The most relevant disturbance predictors identified here were as follows: edge effect, proximity of cattle, minimum fragment age, and median patch size. Interestingly, AGB was efficiently predicted by the proportion of late successional species. We therefore recommend the use of AGB and abundance of late successional species as indicators of human disturbance on high Andean forests.

## Graphical abstract

High Andean forests play a key role in providing ecosystem services for neighboring urban settlements. Here, we aimed to identify and understand the effects of anthropogenic perturbations on diversity, composition, and structure of plant communities, as well as aboveground biomass in high Andean forest remnants in the hinterland of Bogotá. We found that the increase of human‐related disturbance resulted in less phylogenetic diversity and in the phylogenetic clustering of the woody vegetation and in lower aboveground biomass (AGB) values. As to the understory, disturbance was associated with a higher diversity, jointly with a higher phylogenetic dispersion. Interestingly, AGB was efficiently predicted by the proportion of late successional species.

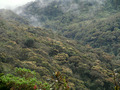

## 1 INTRODUCTION

High Andean tropical montane forests (herein *bosques altoandinos*) can be found between ca. 2,700 and 3,300 m in the Northern Andes, extending from Venezuela to Ecuador, with considerable levels of species diversity and endemism (Gentry & Ortiz, 1993; Girardin et al., 2014; Killeen et al., 2007; Still et al., 1999; Young, 1992). These forests provide vital ecosystem services to the neighboring cities and settlements, such as the regulation of water fluxes (Armenteras et al., 2003; Chaves & Arango, 1998; Linares & Ríos, 2004; Rangel, 2000) or carbon capture and storage (Brown & Kappelle, 2001; Torres et al., 2012).

*Bosques altoandinos* have been subjected to extensive anthropogenic transformation across their natural range. In Colombia, large portions of the forest cover were cleared during the past four centuries and turned into agricultural or residential areas, in order to satisfy the growing demand for resources of an increasing human population (Brown & Kappelle, 2001; Cavelier et al., 2001; Etter et al., 2008; Heath & Binswanger, 1996; Sánchez‐Cuervo et al., 2012; Wassenaar et al., 2007). Such a reduction of forest cover can not only lead to loss of biodiversity but also to a lower structural integrity and resilience of the remaining fragments (Mori et al., 2013). Changes in species composition also go along with shifts in functional diversity and biological interactions (Bovendorp et al., 2019; Diaz & Cabido, 2001; Flynn et al., 2011; Petchey & Gaston, 2002, 2007; Poos et al., 2009; Swenson, 2014). Eventually, this affects ecosystem services (González et al., 2011; Menon et al., 2007; Rangel, 2000; Torres et al., 2012).

In the recent past, forest cover has increasingly been monitored using remote sensing techniques. For the Colombian high Andean forests, this has shown modest signs of recovery in some areas (Calbi et al., 2020; Etter, 2002; Rubiano et al., 2017; Sánchez‐Cuervo et al., 2012; but see Anselm et al., 2018). However, remote sensing cannot detect cryptic forms of forest degradation, such as selective logging or understory grazing. Even plot‐based surveys focusing on trees may not reveal such alterations. Yet, cryptic forest degradation has significant impact on soil erosion, successional dynamics, and regeneration, since understory and epiphytic plants are major drivers of ecosystem functioning (Nilsson & Wardle, 2005). Understanding the effects of anthropogenic disturbance on all major forest components, that is, tree, shrub, understory, and epiphyte layers, is therefore essential to elaborate and implement effective strategies for the sustainable management of these forest ecosystems (Battles et al., 2001; Fahey & Puettmann, 2007; Halpern & Spies, 1995; Roberts & Gilliam, 1995). In addition, multiple predictor and response variables should be analyzed simultaneously to properly address disturbance effects within this complex environment.

One of the best areas to study the impact of human‐induced alterations on *bosques altoandinos* in the northern Andes is the area of Bogotá, the capital of Colombia, which is situated at approximately 2,600 m altitude. With a population of around 9 million inhabitants, Bogotá is by far the largest city in the Andean high montane forest belt, putting tremendous pressure on the surrounding ecosystems. Remnants of high Andean forests near Bogotá are mostly affected by rural activities, which include logging, fires, and agriculture, typically resulting in soil compaction, low fertility, and/or erosion (Armenteras et al., 2003; Linares & Ríos, 2004; Posada & Norden, unpublished results). *Bosques altoandinos* in the surroundings of Bogotá have mostly been studied using phytosociological analysis of plot inventory data (Avella et al., 2014; Cantillo Higuera & Gracia, 2013; Cleef, 1981; Cortés, 2008; Sturm & Rangel, 1985; Van der Hammen, 2008). Beyond such floristically oriented approaches, few studies have addressed the effects of disturbance on these forest ecosystems. Some preliminary research works on forest succession and regeneration were carried out as thesis works (Acuña, 2013; Restrepo Abadia, 2016). In a recent study, Rodríguez‐Alarcón et al. (2018) found a negative effect of forest fragmentation on functional diversity and aboveground biomass, a first indication that more complex parameters such as functional diversity are indeed related to ecosystem services such as carbon storage. However, studies that simultaneously consider multiple disturbance predictors and different plant communities response variables were so far lacking.

According to the available literature, the most relevant disturbance factors, which variation proved to be significantly related to differences in forest species composition or diversity metrics, are as follows: age of forest fragment (Köster et al., 2009; Laurance et al., 2006), proximity to houses or roads and people and livestock density (Ribeiro et al., 2015, 2016), edge effect, and proximity to pastures (Parra Sánchez et al., 2016; Werner & Gradstein, 2009), as well as forest cover fragmentation metrics (Fahrig, 2003; Hertzog et al., 2019; Laurance et al., 2006). Nonetheless, it has not yet been tested whether these factors would be still relevant when a larger number of variables are considered simultaneously. For this reason, we conducted a comprehensive integrated assessment of the potential effects of multiple environmental and disturbance variables on the taxonomic, phylogenetic, and functional diversity of the two main forest layers (tree layer and understory) and on epiphytes cover.

We therefore hypothesized that anthropogenic disturbance as a whole, understood as a composite variable sensu Paine et al. (1998), affects the composition, and aboveground biomass of *bosques altoandinos*, with impacts on community diversity metrics, that is, taxonomic, phylogenetic, and functional diversity. We also hypothesized that our comprehensive analysis would identify significant predictor and response variables other than those found in previous studies. We specifically set out to answer three questions: (a) Which environmental and disturbance variables best explain species diversity and composition of tree and understory layers? (b) What are the effects of *facilitators* (parameters that increase the likeliness of disturbance) and *causes* (direct sources) of disturbance on species diversity, phylogenetic structure, functional diversity, and aboveground biomass? (c) Which vegetation variables are best indicators of disturbance?

## 2 METHODS

### 2.1 Study area

The study area encompasses ca. 4,600 km^2^ within the *Cundiboyacense* high plain in the *Cordillera Oriental* of Colombia, spanning peri‐urban and rural areas of the department of Cundinamarca and the administrative region of the city of Bogotá (Bogotá D.C. or Distrito Capital). The capital region is the most densely populated area of the country, with nearly 9 million inhabitants and approximately 4,500 people per km^2^ (DANE, 2019). The climate is characterized by isothermality with an annual mean temperature of around 14°C and mean annual precipitation between 600 and 1,300 mm. There are two rainy seasons: from April to June and from September to November, with a drier and warmer season from January to March (Anselm et al., 2020; IDEAM, 2007, 2015). The topography is marked by an extended plain, situated at around 2,600 m, which hosts most of the urban and agricultural area, and steep elevation gradients including mountains of up to 4,100 m altitude. Dominant soils in the study area were classified as Andisols (IGAC, 1985; Etter, 2002; Sturm & Rangel, 1985).

Rural areas in the region are highly influenced by the adjacent city of Bogotá and contiguous suburbs. Hence, sparse remnants of original vegetation are intermixed with secondary forests (Cortés, 2008; Rubiano et al., 2017). These remnants are largely dominated, by trees and shrubs in genera such as *Weinmannia*, *Miconia, Clusia, Hesperomeles*, *Clethra, Myrcianthes*, *Myrsine*, *Gaultheria* and *Escallonia*, various genera of Lauraceae, and *Cedrela montana*. Hygrophytic communities with prevalence of *Drimys granadensis* or *Hedyosmum* or higher elevation heliophyte associations of *Gynoxys, Diplostephium*, and *Vallea stipularis* also form part of these ecosystems (Rangel, 2000; Sturm & Rangel, 1985; Van der Hammen, 1998). The forest patches are embedded in a landscape mosaic with cattle pastures and small‐scale cultivation of potatoes (*Solanum tuberosum*), green beans (*Pisum sativum*), and *cubios* (*Tropaeolum tuberosum*). The size of remaining forest fragments is generally small, and their regeneration is threatened by further fragmentation, invasive species, erosion (Linares & Ríos, 2004), and urbanization (Rubiano et al., 2017).

### 2.2 Plot setup

**FIGURE 1:**
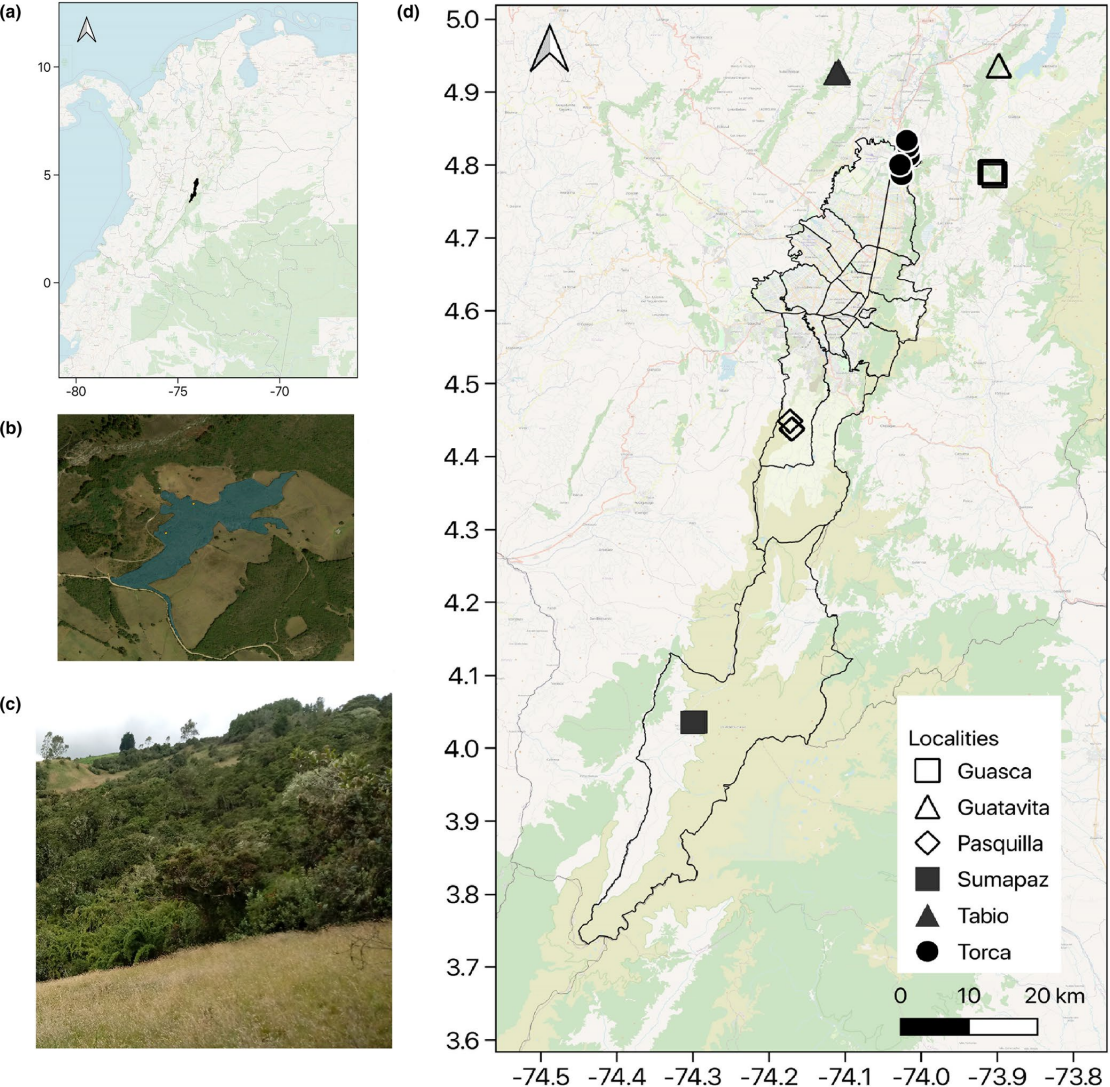
Study area and plot locations. (a) Colombia with Bogotá Capital Department in black; (b) manually vectorized forest fragment in Guatavita; (c) Typical aspect of forest fragment in the study area: (d) Bogotá Capital Department and plot locations. Base map modified from Bing and OSM

Due to the usually small size of forest fragments, we used a plot size of 20 × 20 m (0.04 ha) as established in the framework of the *Rastrojos* project (Acuña, 2013; Hurtado‐Martilletti et al., 2020; Muñoz‐Camacho et al., 2017). We complemented the data from the tree layer assessments of 20 plots obtained from the *Rastrojos* project with data from 12 plots set up and assessed during this study. In addition to the tree layer data, we also assessed the understory layer, and epiphyte cover in the totaling 32 plots, which are located in six administrative regions of Bogotá D.C. and Cundinamarca (Figure 1; Appendix A1). We aimed for a widely scattered position of plots in order to represent the landscape (e.g., including differently inclined slopes). Our sampling design was influenced by the distribution of available and accessible fragments. Plot locations belonged to privately owned protected areas and farms, for which we obtained the required permits of entry from the corresponding owners.

### 2.3 Macro‐environmental variables

For each plot, macro‐environmental variables were compiled from different sources in QGIS 2.18.12 “Las Palmas” (QGIS Development Team, 2018). Altitude, slope, and aspect (northness and eastness) were derived from an Aster Digital elevation model of the study area; for this, ASTGTM2_N04W075, ASTGTM2_N05W075, ASTGTM2_N05W074, and ASTGTM2_N04W074 data products were retrieved from the NASA Land Processes Distributed Active Archive Center (LP DAAC; https://lpdaac.usgs.gov/tools/data‐pool, NASA/METI/AIST/Japan Spacesystems & U.S./Japan ASTER Science Team, 2009). Mean annual precipitation and mean and maximum temperature data for the period 1981–2010 were obtained from the IDEAM meteorological station closest to each plot (http://www.pronosticosyalertas.gov.co/mapas‐graficos‐tiempo‐clima/indicadores‐climatologicos). Mean population density was extracted in two buffers (radius 1 km and 5 km) around the plots from the worldpop database for South America at 1 ha resolution (https://www.worldpop.org, Sorichetta et al., 2015). A complete list of all macro‐environmental variables can be found in the Appendix A2.

### 2.4 Tree and shrub layer assessment

Following the protocol of Hurtado‐Martilletti et al. (2020), for every woody plant with basal diameter > 5 cm (measured at 5 cm from the ground—DAH: Diameter at “ankle” height), we recorded its DAH, DBH and visually estimated tree height, a method that proved to be quite precise for lower canopies such as the ones studied here (Silva et al., 2012). Plant material was collected and identified with the available literature (Gentry & Vasquez, 1993; Trelease & Yuncker, 1950; or webpages: https://plantasdecolombia.com), by comparison with herbarium specimens, digitized specimens available online (JBB: http://herbario.jbb.gov.co; COL: http://www.biovirtual.unal.edu.co/en/collections/search/plants), or with additional help from local experts. Specimens were deposited in the herbarium of the Jardín Botánico de Bogotá José Celestino Mutis (JBB); high‐resolution digital specimen images can be provided upon request; and a plot‐resolved list of vouchers can be found in the Appendix A3.

### 2.5 Understory assessment

In each 20 × 20 m plot, eight 1 × 1 m quadrants (with marked 10 cm subgrids) were placed randomly. All vascular plants, including tree seedlings, were recorded, and mean height and total cover (the sum of all individuals cover) were measured for every species in each quadrant. When available, fertile material was collected and deposited in the JBB. Additionally, cover of bare soil, leaf litter, bryophytes, lichens, and coarse woody debris was visually estimated for every quadrant.

### 2.6 Epiphyte cover

In each plot, we sampled 40 randomly selected trees to estimate the epiphyte cover. Categorical cover classes (ranging from 0 to 3) were assigned to each of five major epiphyte groups (bryophytes, lichens, ferns, bromeliads, and orchids), separately for trunk and canopy branches.

### 2.7 Functional traits and functional diversity

Three leaf functional traits (specific leaf area: SLA; leaf thickness: LT; and leaf dry matter content: LDMC) were measured for each tree species following the protocols provided by Pérez‐Harguindeguy et al., (2013). Five leaves were collected from each of up to three different individuals per species and stored in wet paper for at least 12 hr, then weighted (petiole included). LT was measured with a digital micrometer, and a digital scan of the fresh leaves was taken with a Hewlett‐Packard F4280 scanner. Leaf area was calculated with ImageJ 1.8.0 (Schneider et al., 2012). Leaves were oven‐dried at 60°C until constant weight and weighted; SLA was then calculated as one‐sided area of a fresh leaf divided by its dry mass, expressed in cm^2^/g. LDMC was calculated as the dry mass (mg) divided by its fully hydrated fresh mass (g), and expressed in mg/g. Additionally, wood density (WD) was obtained from Rodríguez‐Alarcón et al. (2018) and the global wood density database (Chave et al., 2009) for all tree species or, depending on availability, at genus or family level estimates, using the R package *biomass* (Réjou‐Méchain et al., 2018) in R Studio (R Core Team, 2018). The traits used to estimate functional diversity were SLA, LDMC, LT, WD, maximum recorded height in the plots, and life form (tree or shrub). The final trait database was completed with data from the *Rastrojos* project including data from published reports (Muñoz‐Camacho et al., 2017) and Posada (unpublished results).

To reduce skewness, traits were log_10_‐transformed and computation of functional divergence, functional dispersion, functional richness, functional evenness, and Rao's quadratic entropy (FDiv, FDis, FRic, FEve and Rao's Q) was performed as indicated in Villéger et al. (2008), using the R package *FD* (Laliberté & Legendre, 2010; Laliberté et al., 2014). We specified “corr + lingoes, m = 3” to reduce dimensionality. Functional diversity (FD) index (Petchey & Gaston, 2002) was calculated as the total branch length of a functional dendrogram generated on a distance matrix of traits with the R function hclust, using the PD function in the R package *picante* (Kembel et al., 2010). We decided to compute functional diversity according to the framework proposed by Mason et al. (2005) and Villéger et al. (2008). The calculated indices provide independent information about the position and relative abundances of species in a multidimensional functional space, allowing for a more detailed examination of the mechanisms linking biodiversity to ecosystem function (Villéger et al., 2008).

### 2.8 Landscape metrics

A Landsat 8 raster was downloaded from the US Geological Survey and processed in QGIS with the *SCP* plugin (Congedo, 2016) to obtain a land cover map. Landscape metrics refer to the size, shape, configuration, number, and position of land‐use patches within a landscape and were obtained for the *forest* class within a 1,000 m diameter buffer zone around the plots with the *LecoS* plugin (Jung, 2013).

Additionally, fragments of forests were manually vectorized and the area was calculated on a prepared Bing aerial map obtained through the *Openlayers* plugin (see Figure 1 for an example). Distance to closest roads was calculated with the *NNJoin* plugin on a shapefile downloaded from the DANE Web site (2018). Also, the type of closest road (main, secondary, or track) was noted. Distances to closest houses or tracks were manually measured on the map. Presence or absence of cattle or active cultivated fields in different buffers (0 m, 50 m, 100 m, or 500 m radius) was surveyed in the field. A complete list of all landscape metrics can be found in the Appendix A2.

Minimum age of the forest cover of each plot was estimated through the visual analysis of 43 aerial pictures of the plot locations acquired from the IGAC (Instituto Geográfico Agustín Codazzi, Bogotá; a detailed list of images can be found in the Appendix A4). The pictures ranged from the year 1940 to 2000 at roughly 10‐year intervals. We searched for available pictures from our plot locations and visually located the plots on the nongeoreferenced images. For each plot, we estimated the minimum age based on the oldest documented continuous occurrence of closed forest. A further analysis of forest cover change during the last seven decades around the study plots, carried out on the same set of aerial pictures, is presented in Calbi et al. (2020).

### 2.9 Community composition and structural variables

Based on the available literature (Cleef, 1981; Cortés, 2008; Cuatrecasas, 1958; Sturm & Rangel, 1985; Van der Hammen, 1998), tree layer species were classified either as late successional slow‐growing, early successional fast‐growing, exotic, or “other” (see Appendix A3 for details). Additionally, understory exotic species cover was calculated. The number of species and the relative proportion of individuals (in case of trees) or the percent cover (in case of the understory) of exotic species were used as indicators of disturbance versus conservation. Variance of tree DBH and height was also computed across all trees within each plot, together with the overall number of tree individuals, stems, stems per tree, and the percentage of large trees (DBH > 30 cm). Mean understory height and cover was calculated, as well as mean epiphytes cover.

The Gini coefficient, a measure of inequality within a distribution widely used in forestry (Bourdier et al., 2016; Latham et al., 1998; Lexerød & Eid, 2006), was calculated in each plot for stem basal areas with the gini function in the R package *reldist* (Handcock, 2016).

### 2.10 Taxonomic and phylogenetic diversity

Alpha‐diversity indices (Shannon's diversity, Simpson's and Pielou's evenness) were computed for each plot with the R package *vegan* (Oksanen et al., 2013). Phylogenetic community structure was assessed on the basis of a published angiosperm supertree (Phylomatic tree R20120829, available at https://github.com/camwebb/tree‐of‐trees/blob/master/megatrees/R20120829.new). First, a regional pool tree was generated with the Phylomatic webtool (Webb & Donoghue, 2005), and then, branch lengths were assigned with the *bladj* algorithm in the software Phylocom 4.2 (Webb et al., 2008), using the *wikstrom.ages* file (Wikström et al., 2001). Phylogenetic diversity (PD), mean pairwise distance (MPD), mean nearest taxon distance (MNTD), and their standardized counterparts (sesPD, sesMPD, and sesMNTD) were calculated for both trees and understory in the R package *picante* (Kembel et al., 2010). Moreover, abundance‐weighted MPD and MNTD were calculated to account for differences in species abundance (Webb et al., 2011). Four species of the Lycopodiaceae had to be removed from the understory regional pool since the family was not included in the used supertree.

The standardized PD metrics express the difference between observed and average value in units of standard deviation (*SD*). Positive values indicate phylogenetic overdispersion (co‐occurring species are more distantly related than expected by chance) and negative values phylogenetic clustering (co‐occurring species are more closely related than expected by chance).

### 2.11 Aboveground biomass

Aboveground tree biomass was calculated with the R package *biomass*. Field measurements of DBH contained less than 5% missing data, so imputation of missing values was performed with the R package *mice* (van Buuren & Groothuis‐Oudshoorn, 2011). To balance the missing data in height measurements, a regional diameter–height model was built in *biomass*. Error propagation was carried out using the AGBmonteCarlo function. Wood density error (errWD) was obtained with the getWoodDensity function as prior values on the uncertainty on wood density values, obtained using the mean sd at the species, genus, and family levels of taxa having at least 10 wood density values in the Global Wood Density database (Réjou‐Méchain et al., 2017). Height error (errH) was calculated as the RSE resulting from the local height–diameter models, as in Réjou‐Méchain et al. (2017), and diameter measurements propagation error (Dpropag) was set to "*chave2004,*" which assigns a standard important error on 5 percent of the measures, and a smaller error on 95 percent of the trees (Réjou‐Méchain et al., 2018).

Mean stand aboveground biomass (AGB) and 95% credibility interval following the error propagation were calculated with the following equation (Chave et al., 2014):

$$\text{AGB}=0.0673*{\left(\text{WD}*H*D^{2}\right)}^{0.976}$$

where AGB = aboveground biomass [kg], WD = wood density [g/cm^3^], *H* = height [m], and *D* = DBH [cm]. Mean AGB per tree was calculated by dividing the total AGB value of each plot by the number of tree individuals.

### 2.12 Data analysis

#### 2.12.1 Drivers of species composition of tree layer and understory

Presence and abundance of all tree, shrub, and liana species were compiled for each plot. Relative abundance was calculated for tree and understory layer mean cover.

Environmental and disturbance‐related variables as well as calculated diversity and biomass metrics were assigned to one of five categories relative to disturbance: *geo‐environmental* = predicting, *causes* = predicting, *facilitators* (parameters that increase the likeliness of disturbance) = predicting, *level* (calculated complex parameters of disturbance outcomes) = response, *indicators* (parameters that indicate directly the degree of disturbance) = response (Appendix A2). For instance, signs of grazing or logging were considered a potential *cause* of disturbance, whereas the nearest distance to a road was considered a potential *facilitator*. Diversity indices and biomass estimation were included in the *level* category.

To filter for dominant variables, we first ordinated the plots based on relative species abundances using nonmetric multidimensional scaling (NMDS) in *vegan,* using the mds function with Bray–Curtis distances, and specifying three as maximum number of axes. Subsequently, we fitted all variables using the envfit function and examined variable ordination scores, in order to identify the variables most strongly correlated with community composition and to assess redundancy. Both predictor and response variables were included in the same analyses, and NMDS was performed separately for tree and understory layer. Second, using Sørensen distances and flexible beta (set to –0.25) as group linkage method (McCune & Mefford, 2015), cluster analysis and subsequently indicator species analysis for each cluster were carried out in PCORD 7 (McCune & Mefford, 2015), in order to further classify community types and their characteristic elements.

Following this preliminary analysis, we determined a subset of variables that correlated with the main axes above a given threshold (*R*_sq_ > 0.35; Table 1) and performed either Kruskal–Wallis or parametric ANOVA, depending on the determined conditional distribution, using the clusters as independent variables and the filtered subsets of variables as response variables.

**TABLE 1:** Variables (only predictors retained) correlating with axes above *R*_Sq_ > 0.35 for trees and understory NMDS

	Variable	*R*_sq_	*p*
Trees	elev	0.84	0.001
	rel_hum	0.81	0.001
	like_adjacencies	0.72	0.001
	splitting_index	0.71	0.001
	patch_cohesion_index	0.68	0.001
	logg	0.63	0.001
	greatest_patch	0.63	0.001
	largest_patch_index	0.62	0.001
	land_cover	0.61	0.001
	landscape_porportion	0.61	0.001
	overall_core	0.59	0.001
	mean_T	0.54	0.001
	landscape_shannon	0.53	0.001
	effective_meshsize	0.52	0.002
	landscape_division	0.52	0.002
	cult_100	0.49	0.001
	cattle	0.49	0.001
	landscape_simpson	0.45	0.003
	cattle_100	0.42	0.003
	age	0.41	0.004
	cattle_50m	0.39	0.006
	road_dist	0.36	0.001
Understory	elev	0.74	0.001
	fragment	0.62	0.001
	overall_core	0.58	0.001
	nn_distance	0.57	0.001
	road_dist	0.55	0.001
	edge_density	0.55	0.001
	edge_lenght	0.55	0.001
	m_DBH	0.54	0.001
	like_adjacencies	0.53	0.001
	landscape_pielou	0.53	0.001
	people_density_1km	0.49	0.001
	landscape_simpson	0.49	0.001
	mAGBT	0.48	0.001
	people_density_5km	0.45	0.001
	effective_meshsize	0.43	0.001
	landscape_division	0.43	0.001
	landscape_shannon	0.43	0.001
	n_stems	0.43	0.001
	land_cover	0.41	0.002
	landscape_porportion	0.41	0.002
	n_trees	0.40	0.001
	cult_500	0.40	0.001
	mean_H	0.39	0.001
	greatest_patch	0.38	0.003
	largest_patch_index	0.38	0.003
	mean_patch	0.38	0.001
	age	0.36	0.001
	cattle_100	0.36	0.002

To verify the presence of spatial autocorrelation in our predictors and responses, we calculated a geographical distance matrix between study plots and performed Moran's I test for all calculated variables. We detected spatial autocorrelation for 24 predictors, but none in our response variables (i.e., diversity metrics).

Finally, partial redundancy analysis (pRDA) was performed in *vegan*, separately for tree and understory layer. To take into account spatial autocorrelation, we fitted a pRDA specifying “locality” as condition, to be able to rule out locality effect on the ordination. The “condition” argument thereby defines partial terms that are fitted before other constraints and can be used to remove the effects of background variables, and their contribution to decomposing inertia (variance) is reported separately (Oksanen et al., 2013). Additionally, we performed Hellinger transformation of our community data as recommended by Legendre and Gallagher (2001). We further selected predictors from the set obtained with the NMDS screening, by checking for correlation (*r* > 0.7), performing Variance Inflation Factor (VIF) analysis (setting the threshold to 10), and then using the *vegan* ordistep function which performs automatic stepwise model building for constrained ordination methods (Oksanen et al., 2013).

#### 2.12.2 General linearized models between main causes and facilitators of disturbance and main response variables

To select meaningful variables to fit our GLMs, we inspected the NMDS and pRDA graph and selected a set of uncorrelated response variables based on the direction of the arrows in the graphs. We then compiled a set of predictor variables that correlated with each selected response and checked for correlation within each set, removing one of the elements in pairs with *r* > 0.7. In parallel, we merged all predictor sets and removed highly correlated and spatially autocorrelated variables. Once a set of consensus predictors was obtained, we conducted a VIF analysis (setting the threshold to 10) and obtained a reduced set of primary and secondary predictors (Table 2). We thus reduced the pool of *geo‐environmental* variables to four, that of *causes* to four, and that of *facilitators* to seven. In addition, we selected response variables for *level,* including diversity metrics and *indicators*.

**TABLE 2:** Retained predictors for GLMs building

**Predictors**		**Responses**	
**Geo‐environmental**		***Tree layer diversity***	
north	northness	TSR	tree species richness
slope	slope	TPielou	tree Pielou's evenness
mean_prec	mean annual precipitation	Tshann	tree Shannon's diversity
mean_T	mean annual temperature	TsesPD	tree standardized phylogenetic diversity
**Causes**		TsesMPDABU	abundance‐weighted trees standardized mean pairwise distance
cult_50m	cultivated fields in 50 m buffer	TsesMNTDABU	abundance‐weighted trees standardized mean nearest taxon distance
cattle	presence of cattle	FDis	Functional Dispersion
logg	logging signs	Feve	Functional Evenness
protected	pretected status	FDiv	Functional Divergence
**Facilitators**		FRic	Functional Richness
path_dist	distance from closest path	AGBplot	plot above‐ground biomass
house_dist	distance from closest house	***Understory diversity***	
track_dist	distance to closest track	HSR	understory species richness
edge	the plot is located at the edge of the fragment	Hpielou	understory Pielou's evenness
age	minimum age of the plot	Hshann	understory Shannon's diversity index
cattle_100	presence of cattle in 100 m buffer	HsesPD	understory standardized phylogenetic diversity
median_patch	median forest patch size in 1 km buffer	HsesMPDABU	abundance‐weighted understory standardized mean pairwise distances
**Indicators**		HsesMNTDABU	abundance‐weighted understory standardized mean nearest taxon distances
n_inv_sp_T	number of invasive species of trees		
n_FST_sp_T	number of fast‐growing species of trees		
n_FST_ind_T	number of fast‐growing species of trees individuals		
%_n_CON_sp_T	% of species of trees associated with conserved forests		
**Level**			
n_large_trees	number of trees with DBH > 30 cm		
n_stems	number of stems		
n_trees	number of trees		
n_sp > 10DBH	number of species with DBH > 10 cm		
***Tree layer diversity***			
FDiv	Functional divergence		
FRic	Functional richness		
FDis	Functional dispersion		
FEve	Functional evenness		
Tshann	Trees Shannon diversity index		
TsesMPD	Trees standardized mean pair distance		
TsesMPDABU	Abundance‐weighted trees standardized mean pairwise distance		
TMNTDABU	Abundance‐weighted trees standardized mean nearest taxon distance		
AGBplot	plot aboveground biomass		

Note

Predictor categories refer to the groups of predictor variables categorized in (Appendix A2).

For each selected response, we identified the best conditional distribution and then performed automated selection of the optimal Generalized Linear Model (GLM) with the regsubsets function in the *leaps* package (Lumley & Lumley, 2013), unifying all groups of predictor variables. We specified a maximum number of predictors of four. Predictors were scaled, and a “*log”* link was specified in the *family* argument.

Thus, our GLMs related separately number of species (trees and understory), species diversity (Shannon and Pielou's indices for trees and understory), abundance‐weighted phylogenetic diversity and structure (trees sesPD, sesMPDABU, sesMNTDABU; understory sesPD, sesMPDABU, sesMNTDABU), functional diversity (FDiv, FRic, FEve, FDis), and aboveground biomass (AGBplot) as response variables with selected explanatory variables among each group of predictors (*geo‐environmental causes* and *facilitators*).

Second, we performed automated selection of the optimal GLMs with AGB, understory number of species, understory Shannon's and Pielou's indices, understory phylogenetic diversity and structure, as response variables and tree diversity indices, *level* and *indicators* of disturbance as sets of secondary predictor variables.

## 3 RESULTS

### 3.1 Plot‐based species inventory of tree and understory layers

#### 3.1.1 Tree layer

We recorded 9,841 tree individuals belonging to 98 taxa. From these, 89 were identified to species level, six to genus, one to family, and two lianas remained unidentified due to lack of leaves, flowers, or fruits required for identification (see the Appendix A3 for the complete list of species and collected herbarium vouchers). Identified taxa belonged to 64 genera and 41 families. The only conifer recorded in the study area was *Podocarpus oleifolia*, and the only tree fern was *Blechnum schomburgkii*.

Asteraceae (14 species), Melastomataceae, Ericaceae, Primulaceae (with 6 species each), Lauraceae, and Rosaceae (5) were found to be the most diverse families in the study area. *Miconia squamulosa* (1,194 individuals) and *Cavendishia bracteata* (1,130) were the most abundant species across the study area, followed by *Weinmannia tomentosa* (805) and *Daphnopsis caracasana* (522).

#### 3.1.2 Understory layer

Overall, 326 understory taxa were recorded, with 266 of them identified to species level, 59 to genus, and one to family level (Appendix A3). Identified taxa belonged to 174 genera and 82 families. Orchidaceae (41 species), Asteraceae (38), and Polypodiaceae (16) were the most diverse families, followed by Piperaceae (13), Bromeliaceae (12), Melastomataceae (11), Dryopteridaceae (10), Ericaceae (9), and Rosaceae (9). Dryopteridaceae, Orchidaceae, Poaceae, Blechnaceae, and Bromeliaceae were the most abundant families.

### 3.2 Plot‐based community ordination (NMDS, cluster analysis and Kruskal–Wallis test/ANOVA)

#### 3.2.1 Tree layer

For the 3D ordination solution, we obtained a final stress value of 0.1160879 after 206 iterations. Visual interpretation of the NMDS graph led to the identification of three main groups. The subsequent cluster analysis revealed three additional groups, which showed deep divergence in the dendrogram (nodes at less than 50% remaining information), totaling six groups/clusters, which were used for the indicator species analysis (see Appendix A5 for further details on the indicator species analysis results).

The NMDS graph (Appendix A6) showed numerous statistically significant axis correlations of environmental variables including elevation, relative humidity, and mean temperature, while the Kruskal–Wallis test and parametric ANOVA showed that floristic differences among all groups were related to elevation (chi‐sq = 25.94, *p* = .0009), mean temperature (chi‐sq = 20.99, *p* = .0008), relative humidity (chi‐sq = 25.71, *p* = .0001), presence of logging (chi‐sq = 17.57, *p* = .0035), presence of cattle (chi‐sq = 21.05, *p* = .0008), presence of cattle in a 50 m buffer (chi‐sq = 15.90, *p* = .0071), presence of cultivated fields in a 100 m buffer (chi‐sq = 23.94, *p* = .0002), Shannon's landscape diversity (*F* = 5.58, *p* = .0013), like adjacencies (chi‐sq = 18.37, *p* = .0025), distance to roads (chi‐sq = 19.45, *p* = .0016), and minimum age of the fragment (chi‐sq = 11.95, *p* = .0355).

The resulting NMDS graphs highlighted some interesting patterns. The NMDS graph of axis 1 versus 2 depicted the variables linked to aboveground biomass (AGB), percentage of late successional species, DBH, height and minimum age on the right hand side, opposite to the variables linked to the number of fast‐growing species of trees, mean exotic species cover in the understory, or to the number of trees and the number of stems in the plots (inverse correlation). In the same plot, the AGB showed high positive correlation with distances to roads, lichen cover in the canopy and mosses cover on the soil and inverse correlation with functional diversity, and Gini coefficient. Moreover, trees abundance‐weighted mean nearest taxon distance (TMNTDABU) lied opposite to the indicators of fragmentation. A complete table of NMDS variable correlation filtered through species abundance can be found in Appendix A7.

#### 3.2.2 Understory layer

For the 3D ordination solution, we obtained a final stress value of 0.1514607 after 20 iterations. Visual grouping within the NMDS graph was not feasible (Appendix A8). The cluster analysis identified five main groups/clusters selecting nodes at less than 20% remaining information. Indicator species analysis did not clearly separate the plot localities from each other (Appendix A5).

Kruskal–Wallis test and parametric ANOVA showed that floristic differences among all groups were related to elevation (chi‐sq = 24.06, *p* = .0001), distance to roads (chi‐sq = 14.23, *p* = .0066), edge density (*F* = 4.93, *p* = .004), presence of cultivated fields in a 100 m buffer (chi‐sq = 23.93, *p* = .0002), mean tree AGB (chi‐sq = 13.69, *p* = .01774), Shannon's landscape diversity (*F* = 3.04, *p* = .0343), people density in a 5 km buffer (chi‐sq = 15.88, *p* = .0032), and presence of cultivated fields in a 500 m buffer (chi‐sq = 8.35, *p* = .0797).

In the understory, elevation again was the most correlated environmental variable with species abundances (see Appendix A7 for details on variables correlation with NMDS axis). In the NDMS graph of axis 1 versus 2, the indicators of fragmentation, together with the presence of cattle and cultivated fields in the vicinity, were located opposite to the indicators of continuous forest cover and most of trees diversity metrics. AGB correlated directly with number of late successional species and distance to paths and tracks, and inversely with the number of fast‐growing species of trees and exotic understory species. Most of understory diversity metrics pointed toward the lower part of the graphs, together with fragmentation indicators and exotic species cover in the understory, number of trees, stems, and fast‐growing species of trees. Understory phylogenetic mean pairwise distances were correlated with AGB.

### 3.3 pRDA

#### 3.3.1 Tree layer

From the set of 25 variables with *R*_sq_ > 0.35 (Table 1), after testing for redundancy, we limited our analysis to a subset of 10 variables: elevation, presence of logging, Shannon's landscape diversity, mean temperature, presence of cattle, presence of cultivated fields in a 100 m buffer, minimum fragment age, distance to roads, and presence of cattle in a 50 m buffer. The ordistep function selected seven of these: elevation, presence of logging, Shannon's landscape diversity, mean temperature, presence of cattle, minimum fragment age, and distance to roads.

**FIGURE 2:**
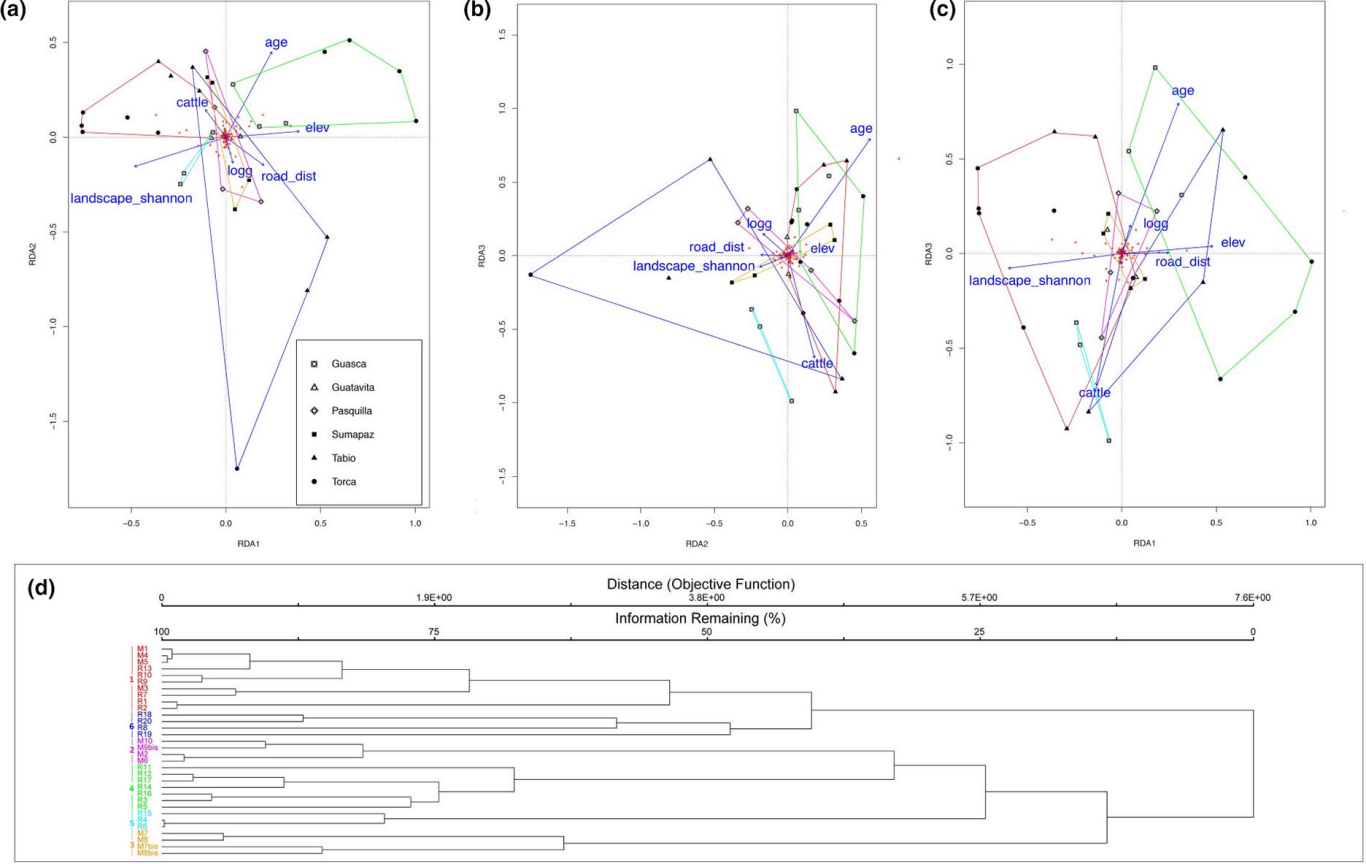
pRDA and Cluster analysis convex hulls of the tree layer. RDA graphs with convex hull volumes of tree layer groups for axis 1–2 (a), 2–3 (b), and 1–3 (c). (d) cluster dendrogram of plots species communities. Group 1 had *Myrcianthes leucoxyla, Viburnum triphyllum*, and *Miconia elaeoides* as statistically significant indicator species and comprised plots from Torca, Tabio, and Guatavita. Group 2 was characterized by *Monticalia pulchella*, *Macleania rupestris,* and *Ilex kunthiana*, and comprised plots exclusively from Pasquilla. Group 3 had *Gaultheria anastomosans*, *Ageratina glyptophlebia*, *Buquetia glutinosa*, *Ageratina boyacensis*, *Berberis glauca,* and *Vaccinium floribundum* as statistically significant indicator species, and comprised exclusively plots form Sumapaz. Group 4 was characterized by *Myrsine coriacea* and *Clusia multiflora* and included plots from Torca and Guasca. Group 5 included *Cavendishia bracteata*, *Diplostephium rosmarinifolium*, *Gaiadendron punctatum,* and *Ulex europaeus,* and comprised only plots from Guasca. Group 6 had *Varronia cylindrostachia* and *Myrsine guianensis* and included plots from Tabio and from Torca. For detailed IVI values and relative *p*‐values refer to the Appendix A5

The pRDA had an *R*_sq_ of 0.23 and adjusted *R*_sq_ of 0.17. The proportional conditional explained variance was 0.45, while the constrained explained variance was 0.24. The unconstrained explained variance was 0.31. Presence of cattle and lower distances to roads were associated with tree layer group 1 which was also positively correlated with Shannon's landscape diversity and negatively with elevation. Group 4 was defined by lower values of Shannon's landscape diversity and was positively correlated with minimum fragment age. Group 5 had some degree of negative correlation with minimum fragment age. Group 6 had an inverse correlation with elevation and minimum fragment age, and was associated with signs of logging, higher Shannon's landscape diversity, and absence of cattle. Groups 2 and 3 were not characterized by any particular association with the ordination variables (Figure 2).

#### 3.3.2 Understory layer

From the set of 38 variables with *R*_sq_ > 0.35 (Table 1), after the assessment of redundancy, we limited our analysis to a subset of 10: elevation, number of trees, edge density, Shannon's landscape diversity, mean tree aboveground biomass (mAGBT), presence of cultivated fields in a 500 m buffer, minimum fragment age, distance to roads, people density in a 5 km buffer, and fragment size. The ordistep function selected seven of these: elevation, edge density, Shannon's landscape diversity, mAGBT, presence of cultivated fields in a 500 m buffer, distance to roads, and people density in a 5 km buffer.

The pRDA had an *R*_sq_ of 0.26 and adjusted *R*_sq_ of 0.11. The proportional conditional explained variance was 0.29, while the constrained explained variance was 0.26. The unconstrained was 0.45.

**FIGURE 3:**
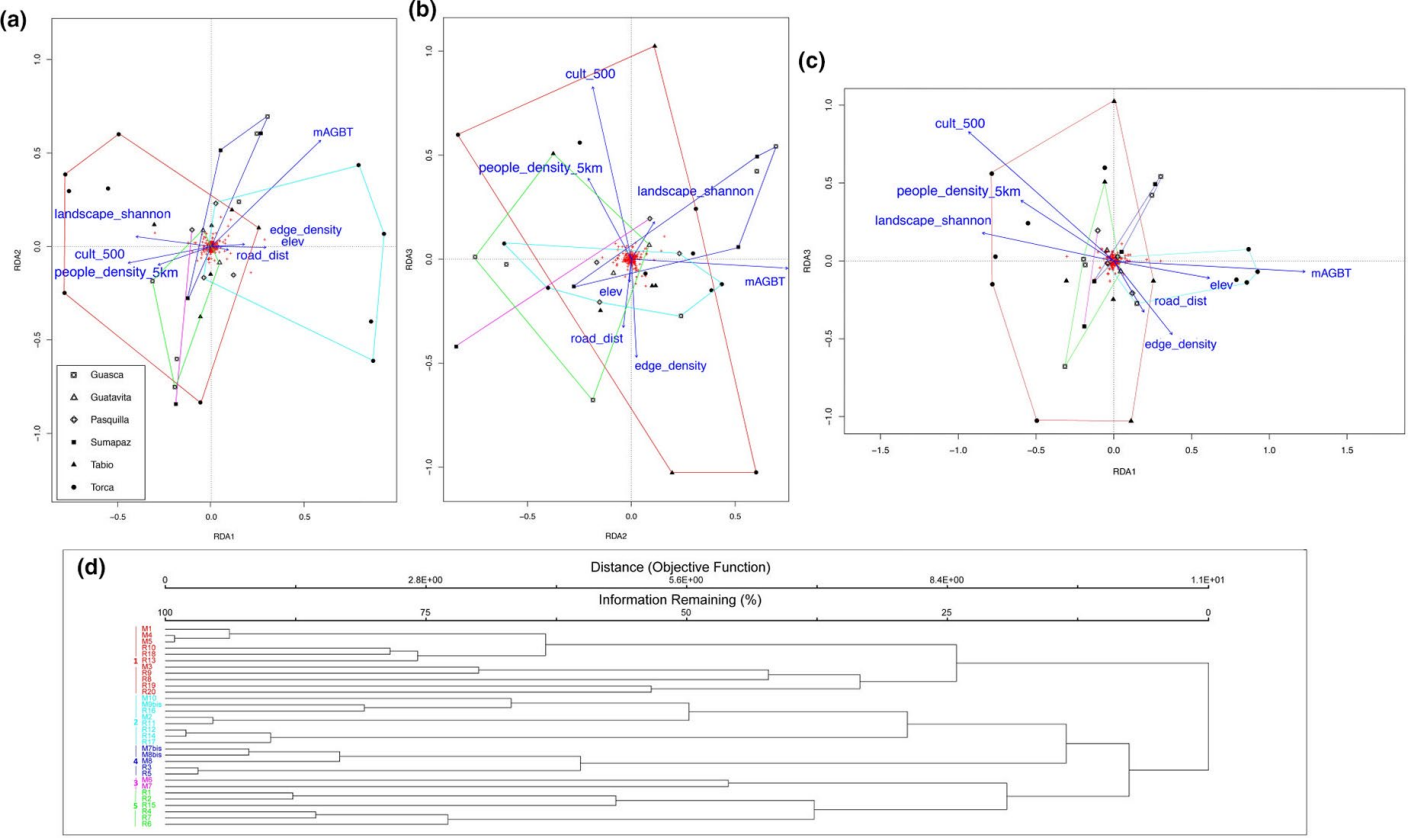
pRDA and Cluster analysis convex hulls of the understory. RDA graphs with convex hull volumes of understory groups for axis 1–2 (a), 2–3 (b), and 1–3 (c). (d) cluster dendrogram of plots species communities. The first group had *Oreopanax incisus* and *Passiflora bogotensis* as indicator species (Appendix A5) and included plots from Torca and Tabio. The second group had *Elaphoglossum lingua* as indicator species, including plots form Pasquilla, Guasca, and Torca. The third group had *Monnina aestuans*, *Peperomia rotundata,* and *Nertera granadensis* among higher valued indicator species. It included only two plots, one from Sumapaz and one from Pasquilla. The fourth group had *Greigia stenolepis* and *Rubus acanthophyllos* among indicator species and comprised plots from Sumapaz and Guasca. The last group had *Ageratina asclepiadea* as indicator species and comprised plots from Guatavita, Guasca, and Tabio. For detailed IVI values and relative *p*‐values refer to the Appendix A5

The results of the pRDA indicated that group 1 was characterized by higher values of Shannon's landscape diversity, lower values for distance from roads, lower elevation, and lower edge density. In contrast with that, group 2 was linked with higher values for distance from roads, higher elevation, lower Shannon's landscape diversity, absence of cultivated fields in a 500 m buffer, and lower population density. Group 3 had lower values of mean tree biomass and higher values of population density. Group 5 was associated with lower values of mean tree biomass. Group 4 was not characterized by any particular association with the ordination variables (Figure 3).

### 3.4 Generalized linear models

A total of 15 primary predictors, 17 secondary predictors, and 17 responses were retained for GLM building (Table 2). Significant variables in GLMs with either a good fit (McFadden *R*_sq_ > 0.2) or a high Nagelkerke value (variance explained > 0.50) are reported below (Tables 3, 4 and 5). A complete table of all fitted GLMs is provided in Table S1. None of the variables associated with epiphytes cover was retained through the variable selection process and analysis.

**TABLE 3:** GLMs of predictors versus responses, showing only GLMs with a good fit

Response	Best model	Variable	Coefficients				Pseudo *R* ^2^	
			Estimate	*SE*	*t* value	*p* value (>|t|)	McFadden	Nagelkerke
Tshann	Tshann ~ slope+mean_T + house_dist+cult_50m	(Intercept)	0.70361	0.02540	27.699	**<2e−16**	**0.752990**	**0.814632**
		slope	−0.08664	0.02698	−3.211	**.0034**		
		mean_T	−0.02686	0.02873	−0.935	.3580		
		house_dist	−0.04705	0.02583	−1.822	.0796		
		cult_50m	−0.04004	0.02870	−1.395	.1744		
Hshann	Hshann ~ track_dist+mean_prec + age+house_dist	(Intercept)	0.89579	0.02215	40.441	**<2e−16**	**0.669964**	**0.759986**
		track_dist	0.06937	0.02916	2.379	**.024680**		
		mean_prec	0.08776	0.02867	3.061	**.004944**		
		age	−0.10445	0.02380	−4.389	**.000157**		
		house_dist	−0.03438	0.02257	−1.523	.139413		
AGB	AGBplot ~ slope+age + cattle+cult_50m	(Intercept)	1.7145	0.07224	23.734	**<2e−16**	0.150415	**0.525516**
		slope	−0.17248	0.07773	−2.219	.035092		
		age	0.31665	0.07803	4.058	**.000379**		
		cattle	0.23182	0.09783	2.370	**.025207**		
		cult_50m	−0.23070	0.09631	−2.395	**.023807**		

Note

Predictor categories refer to the groups of predictor variables categorized in the Appendix A2.

**TABLE 4:** GLMs of secondary predictors versus responses, showing only GLMs with a good fit

Response	Best model	Variable	Coefficients				Pseudo *R* ^2^	
			Estimate	*SE*	*t* value	*p* value (>|*t*|)	McFadden	Nagelkerke
Hshann	Hshann ~ FDis+FRic + n_stems+n_inv_sp_T	(Intercept)	0.89802	0.02507	35.825	**<2e−16**	**0.370602**	0.474078
		FDis	−0.07597	0.03485	−2.180	**.0382**		
		FRic	0.07827	0.03082	2.540	**.0172**		
		n_stems	0.03100	0.02455	1.262	.2176		
		n_inv_sp_T	−0.04740	0.02916	−1.626	.1156		
HsesMPDABU	HsesMPDABU ~ FDiv+FRic + n_sp.10DBH + TsesMPDABU	(Intercept)	0.82655	0.07123	11.605	**5.32e−12**	**0.261149**	**0.581732**
		FDiv	0.24527	0.08248	2.974	**.00613**		
		FRic	−0.26250	0.08501	−3.088	**.00463**		
		n_sp.10DBH	0.12922	0.07900	1.636	.11351		
		TsesMPDABU	0.15706	0.05962	2.634	**.01380**		
AGB	AGBplot ~ TsesMPD+n_large_trees + n_trees+%n_CON_sp_T	(Intercept)	1.7140	0.07467	22.955	**<2e−16**	0.151863	**0.528924**
		TsesMPD	−0.06882	0.11824	−0.582	.56535		
		n_large_trees	0.36350	0.11200	3.246	**.00312**		
		n_trees	0.22454	0.10409	2.157	**.04005**		
		%n_CON_sp_T	0.29929	0.11420	2.621	**.01423**		

Note

Predictor categories refer to the groups of predictor variables categorized in the Appendix A2.

**TABLE 5:** GLMs variables relationships: + indicates a positive relationship and − indicates a negative relationship. Higlighted cells represent GLMs with a good fit

	TSR	Tshann	Tpielou	TsesPD	TsesMPDABU	TsesMNTDABU	HSR	Hshann	Hpielou	HsesPD	HsesMNTDABU	HsesMPDABU	FDis	FDiv	FEve	FRic	AGB
mean_T			−							−							−
mean_prec								+									
north	+																
slope	−	−	−			−						−	−			−	−
logg	−								+						−		
cattle																	+
cult_50m						−	−				+						−
protected				−							−						
edge										−	−	+	+	−		−	
house_dist	−																
cattle_100					−	−					+						
median_patch							−										
age								−	−								+
track_dist								+		−							
n.sp_10DBH											+						
n_trees											+						+
n_large_trees																	+
%n_CON_sp_T																	+
n_FST_ind_T										+	+						
n_stems									+								
FDiv							+					+					
FRic								+		+	−	−					
FDis								−		−							
TsesMPDABU										+		+					
TsesMPD									+								

Tree layer Shannon's diversity decreased with slope. Understory Shannon's diversity increased with distance to tracks, mean precipitation, and tree layer functional richness (FRic), but decreased with minimum age and functional dispersion (FDis). Understory abundance‐weighted phylogenetic mean pairwise distances (HsesMPDABU) increased with functional divergence (FDiv) and tree layer abundance‐weighted phylogenetic mean pairwise distances (TsesMPDABU) and decreased with FRic. Aboveground biomass (AGB) increased with increasing minimum age of the plot and presence of cattle within the plot, and decreased with slope and proximity of cultivated fields. AGB also increased with the number of trees and large trees and with the proportion of late successional species of tree.

#### 3.4.1 Other general trends (from models without a good fit)

Among environmental predictors, slope had a negative effect on FDis, FRic, HsesMPDABU, tree layer species richness (TSR), tree layer Pielou's evenness (Tpielou), tree layer abundance‐weighted mean phylogenetic nearest neighbor distance (TsesMNTDABU). Mean temperature had a negative effect on understory phylogenetic diversity (HsesPD) and Tpielou. Northness had a positive effect on TSR. Among the *causes* predictors, logging had negative effect on functional evenness (FEve) and TSR, and a positive effect on understory layer Pielou's evenness (Hpielou). The presence of cultivated fields in the immediate surrounding of plots (50 m) had a negative effect on HSR and TsesMNTDABU and a positive effect on understory abundance‐weighted mean phylogenetic nearest neighbor distance (HsesMNTDABU). Protection status had a negative effect on HsesMNTDABU and tree layer phylogenetic diversity (TsesPD). As to the *facilitators*, the edge effect was linked with higher values of FDis and HsesMPDABU, and lower values of HsesMNTDABU, HsesPD, FDiv, and FRic. Increasing distance from houses had a negative effect on TSR, while increasing distance from tracks had negative effect on HsesPD. The presence of cattle in a 100 m buffer was linked to higher values of HsesMNTDABU, and lower values of tree layer abundance‐weighted mean phylogenetic pairwise distance (TsesMPDABU) and TsesMNTDABU. Median patch size had negative effect on HSR. Increasing minimum fragment age had a negative effect on HPielou. Coming to the secondary predictors, the number of species with DBH > 10 cm and the number of trees had positive effect on HsesMNTDABU. The number of fast‐growing trees species individuals had a positive effect on HsesMNTDABU and HsesPD. The number of stems had a positive effect on Hpielou. FDiv had a positive effect on HSR. FRic had negative effect on HsesMNTDABU and positive on HsesPD. FDis had a negative effect on HsesPD. Finally, TsesMPDABU had positive effect on HsesPD and TsesMPD had positive effect on Hpielou.

## 4 DISCUSSION

Pressure of urbanization on natural environments and its consequences has been the subject of numerous studies. However, high Andean forests (*bosques altoandinos*) have rarely been investigated in this context. Our study is the first to analyze the role of multiple factors in shaping environmental impact on these forests through urbanization and associated factors in the metropolitan area of Bogotá. However, we are aware of the limitations of this research, which is of rather explorative character and based on data from an area of in total 1.28 ha only. Our sampling design reflects the hurdles of working in a mixed urban–rural matrix, mostly privately owned. Also, having a limited number of plots, we decided to put a stronger emphasis on the variables filtering, to drastically reduce the number of tested hypotheses. Nevertheless, the studied forest fragments belong to several of the localities harboring the highest forest cover within the Capital District and we find the types of high Andean forests covered here to be representative for the hinterland of Bogotá.

Using the composition of natural vegetation as a benchmark, our study plots were dominated by Melastomataceae, Ericaceae, and Asteraceae in the tree layer, which is in accordance with previous work (Cuatrecasas, 1934, 1958; Franco et al., 2010; Torres & Marina, 2016). Bromeliaceae and Orchidaceae were the most diverse families in the understory, coinciding with reports by Cuatrecasas (1934, 1958) and Rangel et al. (2008). Notably, with the exception of Rangel et al. (2008), no recent inventories of the understory were undertaken in the target area prior to this study. The fact that many epiphytic species were found terrestrial in the understory may be due to certain favorable environmental conditions, such as low incidence of light, high humidity, and lower influence of wind than in the canopy (Krömer et al., 2007).

Overall tree species richness of the total area assessed (98) was similar to the 90 taxa reported by Rodríguez‐Alarcón et al. (2018) for an ecologically similar study area near Bogotá. Van der Hammen (1998) reported 50–60 species for 500 m^2^ plots of high Andean forest in the watershed of the Rio Bogotá, 20–30 of which belonged to trees and shrubs. Our own tree species count ranged between 10 and 24, with an average of 16, per 400 m^2^ plot, and Shannon's tree diversity varied between 1.05 and 2.6. Overall, these figures also compare well to those reported for high Andean forest ecosystems (2,300–2,900 m) in Southern Ecuador by Cabrera et al. (2019), who used a higher DBH threshold (10 cm) and obtained about 21 tree species and an average value of 2.44 for Shannon's diversity.

Thus far, only few published studies exist for the target area that aimed at characterizing the various communities of *bosques altoandinos* in terms of species composition. Using a phytosociological approach, Cortés et al. (1999) and Cortés (2008) described the *Myrcianthes leucoxyla‐Miconia squamulosa* community for the internal slopes of the Rio Bogotá watershed, characterized by scarce humidity and low precipitation, with high abundance of *Oreopanax incisus* and conspicuous lianas in the understory. This community corresponds to our tree clusters 1 and 6 and understory clusters 1 and 5. The pRDA further revealed a lower elevation, higher Shannon's landscape diversity, lower minimum fragment age, presence of logging and lower distance to roads as characteristic for this community, supporting the notion that it represents secondary forest, probably developing on patches of abandoned agricultural areas on the slopes surrounding cultivated and farmed plains (Cortés, 2008). Understory cluster 5 was generally found at medium elevations, on small high plains, with a drier climate (Cortés, 2008; Cortés et al., 1999), and in forest patches with generally low values of aboveground biomass.

The *Drimys granadensis‐Weinmannia tomentosa* community is a second *bosque altoandino* subtype (Vargas & Zuluaga, 1980), corresponding to our tree clusters 2 and 4. Cluster 2 is similar to the *Criotoniopsis bogotana*‐*Weinmannia tomentosa* forest subtypes described for elevations between 3,100 and 3,300 m (Cortés, 2008), whereas cluster 4 is found at the slopes and peaks of the watershed of the Río Bogotá between 2,700 and 3,200 m (Cortés, 2008). According to Cortés (2008) and Luteyn (2002), the presence of *Macleania rupestris* in the lower canopy of these communities points toward recent human intervention. This association is known to prefer humid, cold climates and steep grounds; according to our field observations, it is also associated with high lichen and moss cover in the canopy, which prosper in such a relatively high humidity (Batke et al., 2015; Munzi et al., 2014; Wolf, 1993). As shown in the pRDA ordination, it is also linked to low Shannon's landscape diversity, and higher minimum fragment age, probably representing secondary forest fragments approaching the structure of natural forest communities.

Our tree clusters 3 and 5 did not correspond to previously described communities. Cluster 3 was restricted to *bosques altoandinos* near Sumapaz, the largest known paramo on Earth. Characteristic elements of this cluster are families of high elevations such as Asteraceae and Ericaceae (Bach et al., 2007; Cuatrecasas, 1958; Sturm & Rangel, 1985), also typically found in areas subjected to fires or selective logging (Cuatrecasas, 1958). The latter notion is supported by the observed presence of both cattle and cultivated fields in the immediate surrounding, by a high Shannon's landscape diversity, and by the presence of logging, indicating recent and ongoing intervention in the area. Nonetheless, full‐grown individuals of *Weinmannia fagaroides* and *Polylepis quadrijuga* were found in two of the plots of this cluster, together with some young individuals of *Podocarpus oleifolia* and *Berberis glauca* abundant in the lower canopy, and a dense cover of mosses and ferns, which suggests that some small “islands” of mature forest elements were able to persist within the disturbed, secondary forest matrix. Understory cluster 3 did not fit any previously described communities either, but the indicator species of this cluster are known to be either dispersed by birds, for example, *Monnina aestuans* (Romero, 2002) and *Nertera granadensis* (Vargas‐Ríos, 1997), or by small mammals or birds, for example, in the case of the sticky fruits of *Peperomia* (Frenzke et al., 2016). Possibly, this cluster represents a successional understory community mainly dispersed by animals, which prosper in previously disturbed areas, as suggested by the high people density within 5 km radius and relatively low mean tree biomass. Tree cluster 5 was found in the Guasca region only and exhibits features of a disturbed, gap‐filled forest (azonal *páramo*) including the presence of invasive *Ulex europaeus*, which is confirmed by the pRDA correlation with lower minimum fragment age values. Another common species, *Cavendishia bracteata*, has been associated with secondary growth (Cortés, 2008). This cluster had rather low like adjacencies values and average Shannon's landscape diversity and distances to roads, which point to a somehow continued disturbance regime in the past. Indeed, this area, up to the 1990s, used to be an open‐pit limestone mine (Pèrez Sanz de Santamaría,  2013).

Notably, tree and understory communities found in the same plots did not always correspond to the same community's type, which suggests that different types of intervention act differentially on the tree and understory layers. For instance, cattle grazing, erosion, and expansion of edge species will affect the understory at a different pace than the tree layer (Halpern & Lutz, 2013; Millspaugh & Thompson, 2011; Thrippleton et al., 2016).

Our findings support the notion that *bosques altoandinos* in the vicinity of Bogotá are floristically and structurally not homogeneous, resulting in overall high species diversity, especially in the understory, with each of the study sites and plots contributing a portion to this diversity (i.e., high beta diversity). The observed differences in species composition between the study sites, and the high proportion of pRDA‐explained variance that was linked to the “locality” condition, may be determined by topographic variation, which promotes changes in structure, composition, and dynamics of the vegetation, even at small scales in high Andean ecosystems (Homeier et al., 2010; López & Duque, 2010). Our results are similar to a recent study that found substantial differences in species composition between municipalities in the region (Hurtado‐Martilletti et al., 2020), pointing toward the importance of landscape and habitat heterogeneity as a relevant criterion when assessing the impact of urbanization, since each locality may contribute unique elements of diversity not present at other localities, even within close distances. Following up on our first research question, taken aside the effects of local homogenization processes, our data show that plant communities in *bosques altoandinos* are mainly driven by a limited suite of geo‐environmental and disturbance factors, namely: elevation, mean temperature and relative humidity on one hand, and by the presence of cultivated fields and cattle in the immediate sourroundings of the plots, population density, Shannon's landscape diversity, and forest edge density on the other.

The compositionally based clustering of tree and understory communities was largely correlated with both geo‐environmental and disturbance variables, namely, elevation, people density, Shannon's landscape diversity and distance to roads. Mean temperature, relative humidity, logging, and minimum plot age were important factors driving tree species composition, but not the composition of understory species. For the latter, additional variables associated with edge effects, such as the proximity to cultivated fields, edge density, and distance from main roads were relevant. Additionally, mean tree aboveground biomass was a determinant factor in shaping the understory community. These results support the notion of a higher sensitivity of the understory to fragmentation and habitat heterogeneity (Forman & Alexander, 1998; Tyser & Worley, 1992).

Our results show effects of both geo‐environmental parameters and disturbance‐related variables as predictors of both community structure and diversity. Among the geo‐environmental parameters, the negative effects of the increase in slope on tree and understory diversity and aboveground biomass were evident. Slope is related to soil erosion, water drainage, and other unfavorable growth conditions which may act as environmental filters, reducing the number of taxa that can cope with them effectively and may also limit aboveground productivity. Higher mean temperatures were linked to lower tree Pielou's evenness and Understory phylogenetic diversity. This fact could be linked to the higher density of human activities at milder temperatures/lower parts of our study area, which are associated with highly disturbed forest communities, mostly dominated by species as *Miconia squamulosa* or *Cavendishia bracteata,* and host poorer understory communities. Higher precipitation values were linked to higher understory Shannon's diversity, possibly due to increased soil nutrients and moisture and thus by the absence of an environmental filter related to water availability.

With regard to human disturbance predictors, many of the previously identified relevant variables in literature were also selected through our multi‐step analysis, such as minimum age of the forest fragment, distance to houses, edge effect, and presence or proximity of cattle and cultivated fields. People density, on the other hand, showed to be too spatially autocorrelated to be used in our GLMs. Also, among all calculated forest fragmentation metrics, the only one which was selected was (median) forest patch size, already reported to be relevant for plant diversity as an indirect measure of habitat loss in the review of Fahrig (2003). As to the selected responses, tree layer diversity metrics were not particularly sensitive, retrieving only one GLM with a good fit. The correlation between higher distance from houses and forest protection status with lower tree species richness and low phylogenetic diversity was not immediately intuitive, but could be a sign of the deliberate introduction of useful tree species in the vicinity of rural houses, to be harvested for wood or other uses, or of the lack of edge‐related tree species in the interior of protected forest fragments. However, the presence of cattle and cultivated fields in the immediate proximity of plots leading to tree phylogenetic clustering, but on the other hand to understory phylogenetic dispersion, illustrates the disrupting, multi‐layer impact of landscape‐level patchiness and human activities.

Disturbed forests tend to exhibit functional and phylogenetical clustering due to the elimination of entire lineages sensible to disturbance, an effect known as environmental filtering (Chun & Lee, 2018; Gerhold et al., 2015; Kusuma et al., 2018; Mouchet et al., 2010; Ribeiro et al., 2016). Phylogenetic dispersion is expected to be higher in undisturbed, more mature forests than in early successional forests, due to competitive exclusion (Ding et al., 2012; Letcher, 2009; Norden et al., 2012; Purschke et al., 2013). In our study, local, chronic disturbances, such as proximity to farming activities or the presence of cattle in the immediate surroundings, had indeed a negative effect on tree phylogenetic diversity and resulted in phylogenetic clustering, supporting findings by Ribeiro et al. (2015, 2016). Likely, the floristic drift associated with this type of disturbance results in the co‐occurrence of more closely related taxa by decreasing effects of competitive exclusion. On the other hand, the observed increase of phylogenetic dispersion in the understory in close proximity of cattle or cultivated fields may be the result of opportunistic pioneer or exotic species, which introduce different lineages from those associated with more mature forest fragments (Hill & Curran, 2001; Kupfer et al., 2004).

Identified understory diversity metrics with the highest sensitivity to human disturbance were Shannon's diversity and phylogenetic clustering. As suggested by Forman and Alexander (1998) and Tyser and Worley (1992), the number and diversity of understory species were positively related to disturbance‐related variables. Proximity to human activities such as farming and the more recent establishment of forest patches (lower minimum age) fosters generalists or fast‐growing, nutrient‐, and light‐demanding species (Marcantonio et al., 2013). However, at the same time the edge effect promotes less phylogenetic diversity of the understory vegetation, which is in accordance with Ribeiro et al. (2016). This could be explained, in our case, by the fact that ferns and other early diverging taxa diversity tends to diminish toward the edge of a forest fragment to leave place to generalists and agricultural weeds, which can cope better with the site conditions. Larger median forest fragments size also resulted in less understory species, suggesting that recruitment of edge‐related species increment the number of species in smaller forest patches.

The observation that increasing tree functional divergence, and tree phylogenetic dispersion were linked to higher understory phylogenetic dispersion, may indicate that higher trait diversity in the upper stratum allows for more species to colonize the understory. This is partially supported through similar findings by Ampoorter et al. (2014) and Evy et al. (2016), who reported that a multi‐tree species mixture may induce a higher number of understory species, for instance, by modifying environmental conditions relevant to herbaceous plants and seedlings (Vockenhuber et al., 2011). At the same time, functional richness and functional dispersion showed contrasting effects on understory metrics, underlining the multifaceted effect of the multidimensional functional diversity indices. Moreover, the number of trees, large trees, fast‐growing tree individuals, and stems were related to higher understory phylogenetic diversity and dispersion, and to understory Pielou's evenness, confirming that intrastand heterogeneity allows for different understory taxa to thrive due to differences in nutrients, light and water availability (Huebner et al., 1995).

Averaging 149 Mg/ha, the obtained values for aboveground biomass are within the figures reported from other high Andean forest fragments, ranging between 130 and 165 Mg/ha and in some cases up to 640 Mg/ha (Álvarez‐Dávila et al., 2017; Girardin et al., 2014; Rodríguez‐Alarcón et al., 2018). The relatively low mean values obtained here are probably explained by the inclusion of areas characterized by early regeneration stages in several plots. However, our results are higher than those of Moser et al. (2011), who reported 112 Mg/ha for forest plots within a similar elevation range. In regard to our models, AGB seemed to decrease at higher values of slope, which in our study area may relate to eroded soils and drier conditions, supporting a trend that has been reported for relatively moist forests in the Americas (Keith et al., 2009; Stegen et al., 2011), which is perhaps related to the lower soil water content available to sustain photosynthesis (Parton et al., 2012; Stegen et al., 2011), but that can also be a secondary effect of the different rate of agricultural exploitation or forest clearing history between lower and drier and higher and wetter soils in the study area in recent times (Etter et al., 2008; Etter & van Wyngaarden, 2000). Notably, low AGB was linked to the proximity of cultivated fields, suggesting a clear correlation between intervention causing patchy landscapes and lower biomass accumulation. However, the presence of cattle within the plot was linked to higher AGB values. This may be particular to our study area, in which we observed forest fragments with large trees but a much depauperate understory, located in proximity to farms. This is alarming as grazing may interfere with tree species recruitment and stamping may lead to higher soil erosion which in turn will reduce productivity over time in these last standing carbon stock fragments (Nepstad et al., 2002).

The positive correlation that AGB exhibits with the minimum fragment age, and number of trees and large trees, summed to a positive correlation with the percentage of late successional tree species, suggests that AGB is positively influenced by the abundance of slow‐growing species that stock large amount of carbon (Aldana et al., 2017; Álvarez‐Dávila et al., 2017). This finding relates to the question of biomass storage in forest plantations or tree monocultures. Conversely, the increment of environmental stressors in highly fragmented landscapes can increase the mortality of large trees (D'Angelo et al., 2004; Laurance et al., 2000). This promotes the uncontrolled growth of fast‐growing species with lower wood density, which reduces AGB (Berenguer et al., 2014; Chaplin‐Kramer et al., 2015; Laurance & Bierregaard, 1997; de Paula et al., 2011).

In conclusion, the increase of disturbance resulted overall in a negative effect on tree phylogenetic diversity and dispersion. Notably, disturbance affected aboveground biomass negatively. As to the understory, disturbance was associated with more diversity and more phylogenetic dispersion. The *causes* and the *facilitators* category variables were quite efficient in predicting diversity or AGB, among which edge effect, proximity of cattle and cultivated fields, and minimum fragment age appear to be the most important ones.

The plurality of diversity metrics can be difficult to interpret in the light of human disturbance. However, AGB proved to be sensitive to human disturbance and was closely related with the proportion of late successional species. Such indicators could serve as immediate proxies of human disturbance, rather than diversity measures themselves, which have also been shown to react ambiguously to the effects of fragmentation (Fahrig, 2003).

## 5 CONCLUSIONS

In summary, our study on taxonomic, phylogenetic, functional diversity and ABG of high Andean forest underscores the complexity and singularity of interactions between disturbance drivers and plant communities. The main goal of our approach was to test and quantify the alteration of high Andean forest composition, structure, and functioning through human disturbance, testing the effectiveness of known relevant drivers and indicators when a large number of variables are considered simultaneously. We contributed to the characterization of high Andean patterns of tree and understory diversity and local and regional human disturbance, which is usually considered to have a negative effect on native biodiversity and carbon storage. In our case, this fact was confirmed by lower tree layer diversity and a lower ABG in relation to increasing human disturbance, but was however not always apparent through the score of all the diversity metrics that we employed. Decline of AGB and disappearance of the forest ecosystem's late successional species is a warning signal that should impulse protection efforts and restoration measures. Yet, it is also true that the study area has now undergone anthropic disturbance over centuries, with continuous agropastoral activities and subsequent land cover change. In the context of the recovery of forest cover and ecosystem services, then our findings could be interpreted as a positive sign of resilience at a regional scale. Relatively small isolated fragments of high Andean forests can still host high plant diversity and serve as stepping stones or temporary refuges for the local fauna within the rural modified matrix. In this sense, efforts to implement forest connectivity and corridors and to guarantee land‐use continuity even in partially forested areas are priorities that should be taken into account by local decision‐makers. Successful conservation strategies require a sound understanding of community and ecosystem dynamics, and we hope that with the predictors and indicators of disturbance that we pointed out, it will be possible to improve the management strategies for the passive or active restoration and protection of the remaining forest fragments in the study area.

Our results contribute to urgently needed but yet missing baseline knowledge on main drivers of disturbance and its effects on the biodiversity in the study area. However, we strongly recommend that future studies should expand further the established plot network and that more investigations test our results on similar ecosystems to further disentangle the relationship between natural and human‐induced causes of diversity loss and their underlying mechanisms. As shown here, a first approximation can be achieved through an exploratory approach like the one that we employed.

## CONFLICT OF INTEREST

None declared.

## AUTHOR CONTRIBUTIONS

**Mariasole Calbi:** Conceptualization (lead); data curation (lead); formal analysis (lead); investigation (lead); methodology (lead); writing–original draft (lead); writing–review and editing (lead). **Francisco Fajardo‐Gutiérrez:** Data curation (supporting); formal analysis (supporting); investigation (supporting); methodology (supporting); writing–original draft (supporting); writing–review and editing (supporting). **Juan Manuel Posada:** Conceptualization (equal); data curation (equal); investigation (supporting); methodology (lead); project administration (equal); resources (equal); visualization (equal); writing–original draft (supporting); writing–review and editing (supporting). **Robert Lücking:** Conceptualization (equal); data curation (supporting); formal analysis (equal); investigation (equal); methodology (lead); supervision (equal); writing–original draft (equal); writing–review and editing (equal). **Grischa Brokamp:** Funding acquisition (equal); methodology (supporting); project administration (lead); resources (equal); supervision (equal); writing–original draft (equal); writing–review and editing (supporting). **Thomas Borsch:** Conceptualization (supporting); funding acquisition (lead); investigation (equal); methodology (equal); project administration (equal); resources (equal); supervision (lead); writing–original draft (equal); writing–review and editing (supporting).

## Supporting information

Table S1

## Data Availability

The tree layer and understory sampling datasets and the complete table of variables have been submitted to the Dryad digital repository (https://doi.org/10.5061/dryad.z612jm6b5).

## APPENDIX A1

### Plots localities and coordinates

**Table:**

Plot	Locality	Sector	Latitude	Longitude	Elevation
M1	Torca	La Francia	4°47′12.1″	−74°1′34.2″	2,664 m
M2	Pasquilla	Finca Porras	4°26′16.6″	−74°10′09.4″	3,216 m
M3	Torca	La Francia	4°47′11.5″	−74°01′32.8″	2,692 m
M4	Torca	Colegio Nuevo Horizonte	4°48′01.6″	−74°01′42.5″	2,639 m
M5	Torca	Colegio Nuevo Horizonte	4°48′1″	−74°1′41.1″	2,668 m
M6	Pasquilla	Finca Porras	4°26′14.4″	−74°10′14.7″	3,278 m
M7	Sumapaz	Predio Hernan	4°2′10.5″	−74°17′47.6″	3,402 m
M7bis	Sumapaz	Predio Alexandra	4°2′7.7″	−74°18′1.2″	3,395 m
M8	Sumapaz	Predio Hernan	4°2′9.2″	−74°17′51″	3,390 m
M8bis	Sumapaz	Predio Alexandra	4°2′8.0″	−74°18′2.1″	3,387 m
M9bis	Pasquilla	Finca Alveiro	4°26′53.3″	−74°10′21.2″	3,313 m
M10	Pasquilla	Finca Alveiro	4°26′55.9″	−74°10′19.7″	3,307 m
R1	Guatavita	predio Juan	4°56′9.716″	−73°53′54.237″	3,035 m
R2	Guatavita	predio Juan	4°56′12.618″	−73°53′51.825″	3,028 m
R3	Guasca	Encenillo	4°47′20.3172″	−73°54′31.8132″	3,140 m
R4	Guasca	Encenillo	4°47′28.667″	−73°54′25.886″	3,085 m
R5	Guasca	Encenillo	4°47′24.124″	−73°54′31.332″	3,106 m
R6	Guasca	Encenillo	4°47′26.609″	−73°54′25.904″	3,095 m
R7	Tabio	Predio suizo	4°55′40.858″	−74°6′29.194″	2,696 m
R8	Tabio	Predio suizo	4°55′47.149″	−74°6′31.021″	2,707 m
R9	Tabio	Predio suizo	4°55′33.961″	−74°6′47.225″	2,821 m
R10	Tabio	Predio suizo	4°55′31.683″	−74°6′31.579″	2,685 m
R11	Torca	Conjunto floresta	4°48′48.674″	−74°0′58.527″	2,945 m
R12	Torca	Conjunto floresta	4°48′47.937″	−74°0′56.997″	2,965 m
R13	Torca	Conjunto floresta	4°48′31.216″	−74°1′19.178″	2,708 m
R14	Torca	Conjunto floresta	4°48′45.912″	−74°0′58.852″	2,847 m
R15	Guasca	Predio Rosita	4°47′16.5″	−73°54″15.4″	3,056 m
R16	Guasca	Predio Rosita	4°47′05.2″	−73°54′13.8″	3,101 m
R17	Torca	Conjunto portal de Fusca	4°49′30.41″	−74°01′02.49″	3,080 m
R18	Torca	Conjunto portal de Fusca	4°50′00.37″	−74°01′08.96″	2,789 m
R19	Tabio	Predio suizo	4°55′31.79″	−74°06′44.42″	2,736 m
R20	Tabio	Predio suizo	4°55′35.03″	−74°06′40.15″	2,737 m

## APPENDIX A2

### Variables used for the preliminary analysis (NMDS)

**Table:**

Variable acronym	Variable full name	PRED/RESP	Type	Unit	Source
east	eastness = sin(aspect)	PRED	ENV		DEM
elev	elevation	PRED	ENV	m	DEM
mean_prec	mean annual precipitation (1981–2010)	PRED	ENV	mm	IDEAM
mean_T	mean annual temperature (1981–2010)	PRED	ENV	C	IDEAM
north	northness = cos(aspect)	PRED	ENV		DEM
rel_hum	relative humidity (1981–2010)	PRED	ENV	%	IDEAM
sol_rad	solar radiation (1981–2010)	PRED	ENV	kW/m^2^	IDEAM
cattle	cattle inside the plot	PRED	CAU	0/1	Field survey
cattle_50m; cattle_100, cattle_500	cattle in 50, 100 or 500 m from the plot	PRED	CAU	0/1	Field survey
tour	tourism inside the plot	PRED	CAU	0/1	Field survey
logg	logging sings inside the plot	PRED	CAU	0/1	Field survey
other	conservation activities inside the plot	PRED	CAU	0/1	Field survey
protected	protection status	PRED	CAU	0/1	Field survey
age	Minimum age of plot	PRED	FAC	years	Aerial pictures: IGAC
cult_50m; cult_100; cult_500	cultivated fields in 50, 100 and 500 m	PRED	FAC	0/1	Field survey
edge	if the plot was located in the edge = 1, or interior = 0 of fragment	PRED	FAC	0/1	Edge until 50 m
edge_density	Edge density in 1 km buffer	PRED	FAC	m/ha	LC LANDSAT 8 Raster
edge_lenght	Edge length in 1 km buffer	PRED	FAC	km	LC LANDSAT 8 Raster
effective_meshsize	Effective Meshsize in 1 km buffer	PRED	FAC	ha	LC LANDSAT 8 Raster
fractal_dimesion_index	Fractal Dimension Index in 1 km buffer and 500 m	PRED	FAC		LC LANDSAT 8 Raster
fragment	Fragment size	PRED	FAC	km^2^	Bing maps
greatest_patch	Greatest patch area in 1 km buffer and 500 m	PRED	FAC	Ha	LC LANDSAT 8 Raster
land_cover	Land Cover in 1 km buffer	PRED	FAC		LC LANDSAT 8 Raster
landscape_division	Landscape division in 1 km buffer	PRED	FAC		LC LANDSAT 8 Raster
landscape_pielou	Pielou's landscape equitability in 1 km buffer	PRED	FAC		LC LANDSAT 8 Raster
landscape_porportion	Landscape Proportion in 1 km buffer	PRED	FAC	%	LC LANDSAT 8 Raster
landscape_shannon	Shannon,s landscape diversity in 1 km buffer	PRED	FAC		LC LANDSAT 8 Raster
landscape_simpson	Simpson's landscape diversity in 1 km buffer	PRED	FAC		LC LANDSAT 8 Raster
largest_patch_index	Largest Patch Index in 1 km buffer	PRED	FAC	%	LC LANDSAT 8 Raster
like_adjacencies	Like adjacencies in 1 km buffer	PRED	FAC		LC LANDSAT 8 Raster
m_patchshape_ratio	Mean patch shape ratio in 1 km buffer	PRED	FAC		LC LANDSAT 8 Raster
mean_patch	Mean patch area in 1 km buffer	PRED	FAC	ha	LC LANDSAT 8 Raster
median_patch	Median patch area in 1 km buffer	PRED	FAC	ha	LC LANDSAT 8 Raster
n_patches	Number of Patches in 1 km buffer and 500 m	PRED	FAC	n	LC LANDSAT 8 Raster
nn_distance	Euclidean Nearest‐Neighbor Distance in 1 km buffer	PRED	FAC	m	LC LANDSAT 8 Raster
overall_core	Overall Core area in 1 km buffer	PRED	FAC	m	LC LANDSAT 8 Raster
patch_cohesion_index	Patch cohesion index in 1 km buffer	PRED	FAC		LC LANDSAT 8 Raster
patch_density	Patch density in 1 km buffer	PRED	FAC		LC LANDSAT 8 Raster
path_dist	distance from path	PRED	FAC	m	BING map
people_density_1km; people_density_5km	population density in 1 km buffer and 5 km around the plots	PRED	FAC	n/ha	WordPop
road_dist	distance to main roads or not main roads	PRED	FAC	m	DANE
slope	slope in percent	PRED	FAC	%	DEM
smallest_patch	Smallest patch area in 1 km buffer	PRED	FAC	ha	LC LANDSAT 8 Raster
splitting_index	Splitting Index in 1 km buffer and 500 m	PRED	FAC		LC LANDSAT 8 Raster
track_dist	distance from track	PRED	FAC	m	BING map
m_cov_inv_U	mean cover of understory exotic/invasive species	RESP	IND	%	Field survey
m_cov_nat_U	mean cover of native species	RESP	IND	%	Field survey
n.10DBH; n.20DBH	number of individuals of trees with DBH > than 10 or 20 cm	RESP	IND	n	Field survey
n_CON_sp_T; n_CON_ind_T	number of late successional tree species and individuals	RESP	IND	n	Field survey
n_FST_sp_T; n_FST_ind_T	number of fast‐growing tree species and individuals	RESP	IND	n	Field survey
n_inv_sp_T; n_inv_sp_U	number of exotic/invasive tree and understory species	RESP	IND	n	Field survey
n_large_trees	number of large trees (DBH > 30 cm)	RESP	IND	n	Field survey
n_sp.10DBH; n_sp.20DBH	number of species of trees with DBH > than 10 or 20 cm	RESP	IND	n	Field survey
n_stems	number of stems	RESP	IND	n	Field survey
n_trees	number of trees (individuals)	RESP	IND	n	Field survey
stems_tree	mean number of stems per each tree individual	RESP	IND	n	Field survey
%n_CON_sp_T; %n_CON_ind_T	%of total late successional tree species and individuals	RESP	IND	%	Field survey
AGBplot	total aboveground biomass per plot	RESP	LEV	ton	Field survey
DBH_var	DBH variance	RESP	LEV	cm	Field survey
FD	Functional Diversity	RESP	LEV		Field survey
FDis	Functional Dispersion	RESP	LEV		Field survey
FDiv	Functional Evenness	RESP	LEV		Field survey
FEve	Functional Divergence	RESP	LEV		Field survey
FRic	Functional Richness	RESP	LEV		Field survey
Giniun; Giniwe	Gini unweighted and weighted coefficient for basal areas of single trees	RESP	LEV		Field survey
H_var	canopy height variance	RESP	LEV	m	Field survey
m_DBH	mean DBH	RESP	LEV	cm	Field survey
m_H_understory	mean understorey height	RESP	LEV	%	Field survey
mAGBT	mean AGB per tree	RESP	LEV	kg	Field survey
max_H	maximum tree height	RESP	LEV	m	Field survey
mbrioT; mbrioC	mean briophytes cover in trunk and canopy	RESP	LEV	0–3	Field survey
mbroT; mbroC	mean bromeliads cover in trunk and canopy	RESP	LEV	0–3	Field survey
mcobT; mcobC	mean epiphyte cover trunk and canopy	RESP	LEV	0–3	Field survey
morqT; morqC	mean orchids cover in trunk and canopy	RESP	LEV	0–3	Field survey
mliqT; mliqC	mean lichens cover in trunk and canopy	RESP	LEV	0–3	Field survey
mhelT; mhelC	mean ferns cover in trunk and canopy	RESP	LEV	0–3	Field survey
mCWD	mean coarse woody debris cover	RESP	LEV	%	Field survey
mean_H	mean canopy height	RESP	LEV	m	Field survey
mmoss	mean moss cover	RESP	LEV	%	Field survey
msoil	mean soil cover	RESP	LEV	%	Field survey
mundstr	mean understorey cover	RESP	LEV	%	Field survey
TMNTD; HMNTD	Trees and understory mean nearest taxon distance	RESP	LEV		Field survey
TMNTD‐ABU; HMNTD‐ABU	Trees and understory mean nearest taxon distance (abundance weighted)	RESP	LEV		Field survey
TMPD; HMPD	Trees and understory mean pairwise distances	RESP	LEV		Field survey
TMPD‐ABU; HMPD‐ABU	Trees and understory mean pairwise distances (abundance weigthed)	RESP	LEV		Field survey
TPD; HPD	Trees and understory Phylogentic diversity index	RESP	LEV		Field survey
TPIELOU; HPIELOU	Tree and understory Pielou's evenness	RESP	LEV	n	Field survey
TsesMNTD; HsesMNTD	Trees and understory standardized mean nearest taxon distance	RESP	LEV		Field survey
TsesMNTD‐ABU; HsesMNTD_ABU	Trees and understory standardized mean nearest taxon distance (abundance weighted)	RESP	LEV		Field survey
TsesMPD; HsesMPD	Trees and understory standardized mean pairwise distances	RESP	LEV		Field survey
TsesMPD‐ABU; HsesMPD‐ABU	Trees and understory standardized mean pairwise distances (abundance weighted)	RESP	LEV		Field survey
TsesPD; HsesPD	Trees and understory standardized phylogentic diversity index	RESP	LEV		Field survey
Tshann; Hshann	Trees and understory Shannon's diversity index	RESP	LEV		Field survey
Tsimp; Hsimp	Trees and understory Simpson's diversity index	RESP	LEV		Field survey
TSR; HSR	Tree and understory species count	RESP	LEV	n	Field survey
RaoQ	Rao's Q functional diversity	RESP	LEV		Fieldsurvey
%_5;%_all	percent of the 5 more abundant and all tree species found in the understory	RESP	LEV	%	Field survey

Abbreviations: CAU, causes of disturbance; ENV, geo‐environmental; FAC, facilitators of disturbance; IND, indicators of disturbance; LEV, level of disturbance; PRED, predictor; RESP, response.

## APPENDIX A3

### List of taxa of the tree and understory layer and list of plot‐resolved collected vouchers

TREES

**Table:**

Accepted name	Accepted author	Accepted family
*Abatia parviflora*	Ruiz & Pav.	Salicaceae
*Ageratina asclepiadea* ^b^	(L.f.) R.M.King & H.Rob.	Asteraceae
*Ageratina boyacensis*	R.M.King & H.Rob.	Asteraceae
*Ageratina fastigiata*	(Kunth) R.M.King & H.Rob.	Asteraceae
*Ageratina glyptophlebia*	(B.L.Rob.) R.M.King & H.Rob.	Asteraceae
*Ageratina tinifolia*	(Kunth) R.M.King & H.Rob.	Asteraceae
*Aiouea dubia*	(Kunth) Mez	Lauraceae
*Aiouea sp*		Lauraceae
*Alnus acuminata* ^b^	Kunth	Betulaceae
*Baccharis macrantha*	Kunth	Asteraceae
*Baccharis prunifolia*	Kunth	Asteraceae
*Barnadesia spinosa*	L.f.	Asteraceae
*Bejaria resinosa*	Mutis ex L.f.	Ericaceae
*Berberis glauca*	Kunth	Berberidaceae
*Blechnum schomburgkii*	(Klotzsch) C. Chr.	Blechnaceae
*Bocconia frutescens*	L.	Papaveraceae
*Bucquetia glutinosa*	(L. f.) DC.	Melastomataceae
*Carica sp* ^a^		Caricaceae
*Cavendishia bracteata*	(Ruiz & Pav. ex J.St.Hil.) Hoerold	Ericaceae
*Cavendishia nitida*	(Kunth) A.C.Sm.	Ericaceae
*Cedrela montana*	Moritz ex Turcz.	Meliaceae
*Cestrum buxifolium*	Kunth	Solanaceae
*Cestrum sp*		Solanaceae
*Citharexylum sulcatum*	Moldenke	Verbenaceae
*Clethra fagifolia*	Kunth	Clethraceae
*Clethra fimbriata*	Kunth	Clethraceae
*Clethra lanata*	M.Martens & Galeotti	Clethraceae
*Clusia multiflora*	Kunth	Clusiaceae
*Critoniopsis bogotana* ^b^	(Cuatrec.) H.Rob.	Asteraceae
*Croton bogotanus*	Cuatrec.	Euphorbiaceae
*Cybianthus iteoides*	(Benth.) G.Agostini	Primulaceae
*Daphnopsis caracasana*	Meisn.	Thymelaeaceae
*Diplostephium ochraceum*	(Kunth) Nees	Asteraceae
*Diplostephium rosmarinifolium*	(Benth.) Wedd.	Asteraceae
*Drimys granadensis*	L.f.	Winteraceae
*Duranta mutisii*	L.f.	Verbenaceae
*Escallonia myrtilloides*	L.f.	Escalloniaceae
*Escallonia paniculata*	(Ruiz & Pav.) Schult.	Escalloniaceae
*Frangula goudotiana*	(Triana & Planch.) Grubov	Rhamnaceae
*Frangula sphaerosperma*	(Sw.) Kartesz & Gandhi	Rhamnaceae
*Gaiadendron punctatum*	(Ruiz & Pav.) G.Don	Loranthaceae
*Gaultheria anastomosans*	(Mutis ex L.f.) Kunth	Ericaceae
*Hediosmum sp*		Chloranthaceae
*Hesperomeles ferruginea*	(Pers.) Benth.	Rosaceae
*Hesperomeles goudotiana*	(Decne.) Killip	Rosaceae
*Hesperomeles obtusifolia*	(Pers.) Lindl.	Rosaceae
*Ilex kunthiana*	Triana	Aquifoliaceae
*Ilex sp*		Aquifoliaceae
*Lippia hirsuta*	L.f.	Verbenaceae
*Macleania rupestris* ^b^	(Kunth) A.C.Sm.	Ericaceae
*Macrocarpaea glabra*	(L. f.) Gilg	Gentianaceae
*Maytenus laxiflora*	Triana & Planch.	Celastraceae
*MELASTOMATACEAE sp*		Melastomataceae
*Miconia elaeoides*	Naudin	Melastomataceae
*Miconia ligustrina*	(Sm.) Triana	Melastomataceae
*Miconia squamulosa*	Triana	Melastomataceae
*Myrcianthes leucoxyla* ^b^	(Ortega) McVaugh	Myrtaceae
*Myrcianthes rhopaloides*	(Kunth) McVaugh	Myrtaceae
*Morella parvifolia*	(Benth.) Parra‐Os.	Myricaceae
*Morella pubescens*	(Humb. & Bonpl. ex Willd.) Wilbur	Myricaceae
*Myrsine coriacea*	(Sw.) R.Br. ex Roem. & Schult.	Primulaceae
*Myrsine dependens*	(Ruiz & Pav.) Spreng.	Primulaceae
*Myrsine guianensis*	(Aubl.) Kuntze	Primulaceae
*Myrsine latifolia*	(Ruiz & Pav.) Spreng.	Primulaceae
*Myrsine pellucida*	(Ruiz & Pav.) Spreng.	Primulaceae
*Ocotea caesariata*	van der Werff	Lauraceae
*Ocotea heterochroma*	Mez & Sodiro	Lauraceae
*Oreopanax bogotensis*	Cuatrec.	Araliaceae
*Oreopanax incisus* ^b^	(Willd. ex Schult.) Decne. & Planch.	Araliaceae
*Palicourea angustifolia*	Kunth	Rubiaceae
*Palicourea demissa*	Standl.	Rubiaceae
*Palicourea lineariflora*	Wernham	Rubiaceae
*Pentacalia sp*		Asteraceae
*Monticalia pulchella* ^b^	(Kunth) C.Jeffrey	Asteraceae
*Persea ruizii*	J.F.Macbr.	Lauraceae
*Phyllanthus salviifolius*	Kunth	Phyllanthaceae
*Piper bogotense*	C.DC.	Piperaceae
*Podocarpus oleifolius*	D.Don	Podocarpaceae
*Polylepis quadrijuga*	Bitter	Rosaceae
*Prunus buxifolia*	Koehne	Rosaceae
*Psychotria boqueronensis*	Wernham	Rubiaceae
*Sessea corymbiflora*	Goudot ex Rich. Taylor & R. Phillips	Solanaceae
*Solanum cornifolium*	Dunal	Solanaceae
*Symplocos theiformis*	(L. f.) Oken	Symplocaceae
*Tibouchina grossa*	(L. f.) Cogn.	Melastomataceae
*Ulex europaeus* ^a^	L.	Fabaceae
*Vaccinium floribundum*	Kunth	Ericaceae
*Valeriana clematitis*	Kunth	Caprifoliaceae
*Vallea stipularis*	L.f.	Elaeocarpaceae
*Cordia cylindrostachya*	(Ruiz & Pav.) Roem. & Schult.	Boraginaceae
*Verbesina arborea* ^b^	Kunth	Asteraceae
*Viburnum tinoides*	L.f.	Adoxaceae
*Viburnum triphyllum*	Benth.	Adoxaceae
*Weinmannia fagaroides*	Kunth	Cunoniaceae
*Weinmannia tomentosa*	L.f.	Cunoniaceae
*Xylosma spiculifera*	(Tul.) Triana & Planch.	Salicaceae

Late successional.

^a^ Exotic.

^b^ Fast‐growing.

UNDERSTORY

**Table:**

Accepted name	Accepted author	Accepted family
*Acaena cylindristachya*	Ruiz & Pav.	Rosaceae
*Achyrocline satureioides*	(Lam.) DC.	Asteraceae
*Adiantum andicola*	Liebm.	Pteridaceae
*Ageratina asclepiadea*	(L.f.) R.M.King & H.Rob.	Asteraceae
*Ageratina boyacensis*	R.M.King & H.Rob.	Asteraceae
*Ageratina glyptophlebia*	(B.L.Rob.) R.M.King & H.Rob.	Asteraceae
*Ageratina gracilis*	(Kunth) R.M.King & H.Rob.	Asteraceae
*Ageratina tinifolia*	(Kunth) R.M.King & H.Rob.	Asteraceae
*Agrostis perennans*	(Walter) Tuck.	Poaceae
*Alansmia sp*		Grammitidaceae
*Alnus acuminata*	Kunth	Betulaceae
*Alonsoa meridionalis*	(L.f.) Kuntze	Scrophulariaceae
*Anchietea frangulifolia*	(Kunth) Melch.	Violaceae
*Anthoxanthum odoratum* ^a^	L.	Poaceae
*Anthurium caramantae*	Engl.	Araceae
*Arracacia sp*		Apiaceae
*Arrhenatherum elatius* ^a^	(L.) P.Beauv. ex J.Presl & C.Presl.	Poaceae
*Asplenium cladolepton*	Fée	Aspleniaceae
*Asplenium monanthes*	L.	Aspleniaceae
*Asplenium praemorsum*	Sw.	Aspleniaceae
*Asplenium radicans*	L.	Aspleniaceae
*Asplundianthus densus*	(Benth.) R.M.King & H.Rob.	Asteraceae
*ASTERACEAE sp*		Asteraceae
*Athyrium filix‐femina*	(L.) Roth	Woodsiaceae
*Baccharis bogotensis*	Kunth	Asteraceae
*Baccharis latifolia*	(Ruiz & Pav.) Pers.	Asteraceae
*Baccharis lehmannii*	Klatt	Asteraceae
*Baccharis macrantha*	Kunth	Asteraceae
*Barnadesia spinosa*	L.f.	Asteraceae
*Bejaria resinosa*	Mutis ex L. f.	Ericaceae
*Berberis glauca*	Kunth	Berberidaceae
*Berberis goudotii*	Triana & Planch.	Berberidaceae
*Bidens rubifolia*	Kunth	Asteraceae
*Blechnum cordatum*	(Desv.) Hieron.	Blechnaceae
*Blechnum loxense*	(Kunth) Hook. ex Salomon	Blechnaceae
*Blechnum occidentale*	L.	Blechnaceae
*Blechnum schomburgkii*	(Klotzsch) C. Chr.	Blechnaceae
*Boehmeria cylindrica*	(L.) Sw.	Urticaceae
*Boehmeria sp*		Urticaceae
*Bomarea multiflora*	(L. f.) Mirb.	Alstroemeriaceae
*Bomarea sp*		Alstroemeriaceae
*Botrychium virginianum*	(L.) Sw.	Ophioglossaceae
*Bucquetia glutinosa*	(L. f.) DC.	Melastomataceae
*Calamagrostis effusa*	(Kunth) Steud.	Poaceae
*Calceolaria microbefaria*	Kraenzl.	Calceolariaceae
*Campyloneurum angustifolium*	(Sw.) Fée	Polypodiaceae
*Campyloneurum latum*	T. Moore	Polypodiaceae
*Capsella bursa‐pastoris*	(L.) Medik.	Brassicaceae
*Cardamine ovata*	Benth.	Brassicaceae
*Carex pichinchensis*	Kunth	Cyperaceae
*Carex sp*		Cyperaceae
*Castilleja fissifolia*	L.f.	Orobanchaceae
*Cavendishia bracteata*	(Ruiz & Pav. ex J.St.Hil.) Hoerold	Ericaceae
*Cedrela montana*	Moritz ex Turcz.	Meliaceae
*Cestrum buxifolium*	Kunth	Solanaceae
*Chaetolepis lindeniana*	(Naudin) Triana	Melastomataceae
*Chromolaena bullata*	(Klatt) R.M.King & H.Rob.	Asteraceae
*Chromolaena leivensis*	(Hieron.) R.M.King & H.Rob.	Asteraceae
*Chromolaena perglabra*	(B.L.Rob.) R.M.King & H.Rob.	Asteraceae
*Chromolaena scabra*	(L.f.) R.M.King & H.Rob.	Asteraceae
*Chromolaena sp1*		Asteraceae
*Chromolaena sp2*		Asteraceae
*Chusquea scandens*	Kunth	Poaceae
*Citharexylum sulcatum*	Moldenke	Verbenaceae
*Clematis dioica*	L.	Ranunculaceae
*Clematis haenkeana*	C.Presl	Ranunculaceae
*Clethra fimbriata*	Kunth	Clethraceae
*Clusia multiflora*	Kunth	Clusiaceae
*Erigeron canadensis* ^a^	L.	Asteraceae
*Cortaderia nitida*	(Kunth) Pilg.	Poaceae
*Cranichis ciliata*	Kunth	Orchidaceae
*Cranichis sp*		Orchidaceae
*Critoniopsis bogotana*	(Cuatrec.) H.Rob.	Asteraceae
*Croton bogotanus*	Cuatrec.	Euphorbiaceae
*Cuphea hyssopifolia* ^a^	Kunth	Lythraceae
*Cyperus sp*		Cyperaceae
*Cystopteris fragilis*	(L.) Bernh.	Cystopteridaceae
*Daphnopsis caracasana*	Meisn.	Thymelaeaceae
*Digitalis purpurea* ^a^	L.	Plantaginaceae
*Lycopodium thyoides*	Humb. & Bonpl. ex Willd.	Lycopodiaceae
*Diplostephium floribundum*	(Benth.) Wedd.	Asteraceae
*Diplostephium ochraceum*	(Kunth) Nees	Asteraceae
*Diplostephium rosmarinifolium*	(Benth.) Wedd.	Asteraceae
*Diplostephium rosmarinifolium*	(Benth.) Wedd.	Asteraceae
*Drimys granadensis*	L.f.	Winteraceae
*Dryopteris sp*		Dryopteridaceae
*Duranta mutisii*	L.f.	Verbenaceae
*Elaphoglossum cuspidatum*	(Willd.) T. Moore	Dryopteridaceae
*Elaphoglossum engelii*	(H. Karst.) Christ	Dryopteridaceae
*Elaphoglossum gayanum*	(Fée) T. Moore	Dryopteridaceae
*Elaphoglossum latifolium*	(Sw.) J. Sm.	Dryopteridaceae
*Elaphoglossum lindenii*	(Bory ex Fée) T. Moore	Dryopteridaceae
*Elaphoglossum lingua*	(C. Presl) Brack.	Dryopteridaceae
*Elaphoglossum minutum*	(Pohl ex Fée) T. Moore	Dryopteridaceae
*Elaphoglossum sp*		Dryopteridaceae
*Elleanthus aurantiacus*	(Lindl.) Rchb.f.	Orchidaceae
*Elleanthus maculatus*	(Lindl.) Rchb.f.	Orchidaceae
*Elleanthus purpureus*	(Rchb.f.) Rchb.f.	Orchidaceae
*Elleanthus sp*		Orchidaceae
*Epidendrum caesaris*	Hágsater & E.Santiago	Orchidaceae
*Epidendrum cylindraceum*	Lindl.	Orchidaceae
*Epidendrum excisum*	Lindl.	Orchidaceae
*Epidendrum moritzii*	Rchb.f.	Orchidaceae
*Epidendrum scutella*	Lindl.	Orchidaceae
*Epidendrum sisgaense*	Hágsater	Orchidaceae
*Epidendrum sp1*		Orchidaceae
*Epidendrum sp2*		Orchidaceae
*Epidendrum sp3*		Orchidaceae
*Epidendrum sp4*		Orchidaceae
*Equisetum bogotense*	Kunth	Equisetaceae
*Eriosorus flexuosus*	(Kunth) Copel.	Pteridaceae
*Escallonia myrtilloides*	L.f.	Escalloniaceae
*Espeletiopsis corymbosa*	(Humb. & Bonpl.) Cuatrec.	Asteraceae
*Faramea sp*		Rubiaceae
*Fernandezia crystallina*	(Lindl.) M.W.Chase	Orchidaceae
*Fernandezia sanguinea*	(Lindl.) Garay & Dunst.	Orchidaceae
*Fragaria vesca* ^a^	L.	Rosaceae
*Frangula goudotiana*	(Triana & Planch.) Grubov	Rhamnaceae
*Frangula sp*		Rhamnaceae
*Frangula sphaerosperma*	(Sw.) Kartesz & Gandhi	Rhamnaceae
*Fuchsia boliviana* ^a^	Carrière	Onagraceae
*Fuchsia magellanica* ^a^	Lam.	Onagraceae
*Fuchsia paniculata* ^a^	Lindl.	Onagraceae
*Gaiadendron punctatum*	(Ruiz & Pav.) G.Don	Loranthaceae
*Galianthe bogotensis*	(Kunth) E.L.Cabral & Bacigalupo	Rubiaceae
*Galium ascendens*	Willd. ex Spreng.	Rubiaceae
*Galium hypocarpium*	(L.) Endl. ex Griseb.	Rubiaceae
*Gnaphalium americanum*	Mill.	Asteraceae
*Gaultheria anastomosans*	(Mutis ex L.f.) Kunth	Ericaceae
*Gaultheria erecta*	Vent.	Ericaceae
*Geissanthus andinus*	Mez	Primulaceae
*Geranium holosericeum*	Willd. ex Spreng.	Geraniaceae
*Greigia stenolepis*	L.B.Sm.	Bromeliaceae
*Habenaria sp*		Orchidaceae
*Hedyosmum racemosum*	(Ruiz & Pav.) G.Don	Chloranthaceae
*Heppiella ulmifolia*	(Kunth) Hanst.	Gesneriaceae
*Hesperomeles goudotiana*	(Decne.) Killip	Rosaceae
*Hesperomeles obtusifolia*	(Pers.) Lindl.	Rosaceae
*Hieracium avilae*	Kunth	Asteraceae
*Huperzia hippuridea*	(Christ) Holub	Lycopodiaceae
*Hydrocotyle bonplandii*	A.Rich.	Araliaceae
*Hydrocotyle gunnerifolia*	Wedd.	Araliaceae
*Hydrocotyle tenerrima*	Rose ex Mathias	Araliaceae
*Hymenophyllum myriocarpum*	Hook.	Hymenophyllaceae
*Hypericum juniperinum*	Kunth	Hypericaceae
*Hypochaeris radicata* ^a^	L.	Asteraceae
*Ilex kunthiana*	Triana	Aquifoliaceae
*Ilex sp*		Aquifoliaceae
*Jungia ferruginea*	L.f.	Asteraceae
*Lantana camara*	L.	Verbenaceae
*Lantana rugosa*	Thunb.	Verbenaceae
*Lepanthes gargantua*	Rchb.f.	Orchidaceae
*Lepidaploa canescens*	(Kunth) Cass.	Asteraceae
*Luzula gigantea*	Desv.	Juncaceae
*Lycopodium clavatum*	L.	Lycopodiaceae
*Lycopodium jussiaei*	Desv. ex Poir.	Lycopodiaceae
*Macleania rupestris*	(Kunth) A.C.Sm.	Ericaceae
*Macrocarpaea glabra*	(L. f.) Gilg	Gentianaceae
*Malaxis crispifolia*	(Rchb.f.) Kuntze	Orchidaceae
*Malaxis sp*		Orchidaceae
*Matelea mutisiana*	Morillo	Apocynaceae
*Maxillariella graminifolia*	(Kunth) M.A.Blanco & Carnevali	Orchidaceae
*Maxillaria sp*		Orchidaceae
*Ctenopteris flabelliformis*	(Poir.) J. Sm.	Polypodiaceae
*Melpomene moniliformis*	(Lag. ex Sw.) A.R. Sm. & R.C. Moran	Polypodiaceae
*Miconia elaeoides*	Naudin	Melastomataceae
*Miconia latifolia*	(D. Don) Naudin	Melastomataceae
*Miconia ligustrina*	(Sm.) Triana	Melastomataceae
*Miconia micropetala*	Cogn.	Melastomataceae
*Miconia squamulosa*	Triana	Melastomataceae
*Miconia theizans*	(Bonpl.) Cogn.	Melastomataceae
*Monnina aestuans*	(L.f.) DC.	Polygalaceae
*Monnina fastigiata*	(Bonpl.) DC.	Polygalaceae
*Monochaetum bonplandii*	(Humb. & Bonpl.) Naudin	Melastomataceae
*Monochaetum myrtoideum*	Naudin	Melastomataceae
*Morella parvifolia*	(Benth.) Parra‐Os.	Myricaceae
*Munnozia senecionidis*	Benth.	Asteraceae
*Myrcianthes leucoxyla*	(Ortega) McVaugh	Myrtaceae
*Myrsine coriacea*	(Sw.) R.Br. ex Roem. & Schult.	Primulaceae
*Myrsine dependens*	(Ruiz & Pav.) Spreng.	Primulaceae
*Myrsine guianensis*	(Aubl.) Kuntze	Primulaceae
*Myrsine sp*		Primulaceae
*Nertera granadensis*	(Mutis ex L.f.) Druce	Rubiaceae
*Niphogeton*		Apiaceae
*Ocotea heterochroma*	Mez & Sodiro	Lauraceae
*Ocotea longifolia*	Kunth	Lauraceae
*Oligactis sessiliflora*	(Kunth) H.Rob. & Brettell	Asteraceae
*Oreopanax bogotensis*	Cuatrec.	Araliaceae
*Oreopanax incisus*	(Willd. ex Schult.) Decne. & Planch.	Araliaceae
*Oreopanax mutisianus*	(Kunth) Decne. & Planch.	Araliaceae
*Orthrosanthus chimboracensis*	(Kunth) Baker	Iridaceae
*Oxalis acetosella* ^a^	L.	Oxalidaceae
*Oxalis corniculata* ^a^	L.	Oxalidaceae
*Oxalis medicaginea*	Kunth	Oxalidaceae
*Oxalis spiralis*	Ruiz & Pav. ex G.Don	Oxalidaceae
*Oxalis tuberosa*	Molina	Oxalidaceae
*Palicourea angustifolia*	Kunth	Rubiaceae
*Palicourea lineariflora*	Wernham	Rubiaceae
*Panicum sp*		Poaceae
*Paspalum bonplandianum*	Flüggé	Poaceae
*Passiflora adulterina*	L. f.	Passifloraceae
*Passiflora bogotensis*	Benth.	Passifloraceae
*Passiflora capsularis*	L.	Passifloraceae
*Passiflora sp*		Passifloraceae
*Passiflora tripartita*	(Juss.) Poir.	Passifloraceae
*Pecluma divaricata*	(E. Fourn.) Mickel & Beitel	Polypodiaceae
*Pecluma paradiseae*	(Langsd. & Fisch.) M.G. Price	Polypodiaceae
*Pecluma sp*		Polypodiaceae
*Pentacalia nitida*	(Kunth) Cuatrec.	Asteraceae
*Monticalia pulchella*	(Kunth) C.Jeffrey	Asteraceae
*Peperomia alibacophylla*	Trel. & Yunck.	Piperaceae
*Peperomia dendrophila*	Schltdl.	Piperaceae
*Peperomia arthurii*	Trel. & Yunck.	Piperaceae
*Peperomia emarginulata*	C.DC.	Piperaceae
*Peperomia galioides*	Kunth	Piperaceae
*Peperomia glabella*	(Sw.) A.Dietr.	Piperaceae
*Peperomia hartwegiana*	Miq.	Piperaceae
*Peperomia microphylla*	Kunth	Piperaceae
*Peperomia rotundata*	Kunth	Piperaceae
*Peperomia suratana*	Trel. & Yunck.	Piperaceae
*Pernettya sp*		Ericaceae
*Gaultheria myrsinoides*	Kunth	Ericaceae
*Persea ruizii*	J.F.Macbr.	Lauraceae
*Phenax rugosus*	(Poir.) Wedd.	Urticaceae
*Phyllanthus salviifolius*	Kunth	Phyllanthaceae
*Physalis peruviana* ^a^	L.	Solanaceae
*Pilea alsinifolia*	Wedd.	Urticaceae
*Pilea goudotiana*	Wedd.	Urticaceae
*Pilea lindeniana*	Wedd.	Urticaceae
*Pilea sp*		Urticaceae
*Piper artanthe*	C.DC.	Piperaceae
*Piper bogotense*	C.DC.	Piperaceae
*Piper marginatum*	Jacq.	Piperaceae
*Plagiogyria pectinata*	(Liebm.) Lellinger	Plagiogyriaceae
*Pleopeltis macrocarpa*	(Bory ex Willd.) Kaulf.	Polypodiaceae
*Pleopeltis sp 1*		Polypodiaceae
*Pleopeltis sp 2*		Polypodiaceae
*Pleopeltis sp 3*		Polypodiaceae
*Pleurothallis lindenii*	Lindl.	Orchidaceae
*Pleurothallis linguifera*	Lindl.	Orchidaceae
*Podocarpus oleifolius*	D.Don	Podocarpaceae
*Polystichum lehmannii*	Hieron.	Dryopteridaceae
*Ponthieva similis*	C.Schweinf.	Orchidaceae
*Prunus sp*		Rosaceae
*Psychotria boqueronensis*	Wernham	Rubiaceae
*Pteridium aquilinum*	(L.) Kuhn	Dennstaedtiaceae
*Pteris muricata*	Hook.	Pteridaceae
*Rhynchospora macrochaeta*	Steud. ex Boeckeler	Cyperaceae
*Rhynchospora nervosa*	(Vahl) Boeckeler	Cyperaceae
*Rhynchospora sp*		Cyperaceae
*Rubus floribundus*	Kunth	Rosaceae
*Rubus wardii*	Merr.	Rosaceae
*Rubus sp*		Rosaceae
*Rubus ulmifolius*	Schott	Rosaceae
*Salvia sp*		Lamiaceae
*Sauvagesia erecta*	L.	Ochnaceae
*Serpocaulon eleutherophlebium*	(Fée) A.R. Sm.	Polypodiaceae
*Serpocaulon lasiopus*	(Klotzsch) A.R. Sm.	Polypodiaceae
*Serpocaulon levigatum*	(Cav.) A.R. Sm.	Polypodiaceae
*Serpocaulon sp*		Polypodiaceae
*Serpocaulon sessilifolium*	(Desv.) A.R. Sm.	Polypodiaceae
*Setaria italica* ^a^	(L.) P.Beauv.	Poaceae
*Siphocampylus brevicalyx*	E.Wimm.	Campanulaceae
*Smallanthus pyramidalis*	(Triana) H.Rob.	Asteraceae
*Smilax cuspidata*	Duhamel	Smilacaceae
*Smilax sp 1*		Smilacaceae
*Smilax sp 2*		Smilacaceae
*Smilax tomentosa*	Kunth	Smilacaceae
*Solanum caripense*	Dunal	Solanaceae
*Solanum cornifolium*	Dunal	Solanaceae
*Solanum pseudocapsicum*	L.	Solanaceae
*Solanum sp 1*		Solanaceae
*Solanum sp 2*		Solanaceae
*Sphyrospermum buxifolium*	Poepp. & Endl.	Ericaceae
*Stachys arvensis* ^a^	(L.) L.	Lamiaceae
*Stelis argentata*	Lindl.	Orchidaceae
*Stelis galeata*	(Lindl.) Pridgeon & M.W.Chase	Orchidaceae
*Stelis pulchella*	Kunth	Orchidaceae
*Stelis pusilla*	Kunth	Orchidaceae
*Stelis sp 1*		Orchidaceae
*Stelis sp 2*		Orchidaceae
*Stelis sp 3*		Orchidaceae
*Stelis sp 4*		Orchidaceae
*Stelis sp 5*		Orchidaceae
*Stelis sp 6*		Orchidaceae
*Stelis sp 7*		Orchidaceae
*Stelis sp 8*		Orchidaceae
*Stelis sp 9*		Orchidaceae
*Stenorrhynchos speciosum*	(Jacq.) Rich.	Orchidaceae
*Styrax sp*		Styracaceae
*Symplocos lucida*	(Thunb.) Siebold & Zucc.	Symplocaceae
*Thelypteris rudis*	(Kunze) Proctor	Thelypteridaceae
*Tibouchina grossa*	(L. f.) Cogn.	Melastomataceae
*Tigridia pavonia* ^a^	(L.f.) DC.	Iridaceae
*Tillandsia biflora*	Ruiz & Pav.	Bromeliaceae
*Tillandsia complanata*	Benth.	Bromeliaceae
*Tillandsia elongata*	Kunth	Bromeliaceae
*Tillandsia sp 1*		Bromeliaceae
*Tillandsia sp 2*		Bromeliaceae
*Tillandsia sp 3*		Bromeliaceae
*Tillandsia sp 4*		Bromeliaceae
*Tillandsia sp 5*		Bromeliaceae
*Tillandsia sp 6*		Bromeliaceae
*Tillandsia sp 7*		Bromeliaceae
*Tillandsia sp 8*		Bromeliaceae
*Tradescantia sp 1* ^a^		Commelinaceae
*Tradescantia sp 2* ^a^		Commelinaceae
*Ulex europaeus* ^a^	L.	Fabaceae
*Uncinia hamata*	(Sw.) Urb.	Cyperaceae
*Vaccinium floribundum*	Kunth	Ericaceae
*Valeriana clematitis*	Kunth	Caprifoliaceae
*Vallea stipularis*	L.f.	Elaeocarpaceae
*Cordia cylindrostachya*	(Ruiz & Pav.) Roem. & Schult.	Boraginaceae
*Villanova oppositifolia*	Lag.	Asteraceae
*Viburnum tinoides*	L.f.	Adoxaceae
*Viburnum triphyllum*	Benth.	Adoxaceae
*Weinmannia fagaroides*	Kunth	Cunoniaceae
*Weinmannia tomentosa*	L.f.	Cunoniaceae
*Xylosma spiculifera*	(Tul.) Triana & Planch.	Salicaceae

^a^ Exotic or mainly cultivated.

LIST OF PLOT‐RESOLVED COLLECTED VOUCHERS

**Table:**

Collection number (MSC)	Locality	Plot	Species
1	Torca, La Francia	M1	*Ocotea heterocroma*
2	Torca, La Francia	M1	*Daphnopsis caracasana*
3	Torca, La Francia	M1	*Oligactis volubilis*
4	Torca, La Francia	M1	*Oligactis volubilis*
5	Torca, La Francia	M1	*Viburnum tinoides*
6	Torca, La Francia	M1	*Daphnopsis caracasana*
7	Torca, La Francia	M1	*Palicourea (angustifolia)*
8	Torca, La Francia	M1	*Oreopanax incisus*
9	Torca, La Francia	M1	*Daphnopsis caracasana*
10	Torca, La Francia	M1	*Bejaria aestuans*
11	Torca, La Francia	M1	*Piper arthanthe*
12	Torca, La Francia	M1	*Daphnopsis caracasana*
13	Torca, La Francia	M1	*Miconia squamulosa*
14	Torca, La Francia	M1	*Viburnum tryphyllum*
15	Torca, La Francia	M1	*Macleania rupestris*
16	Torca, La Francia	M1	*Daphnopsis caracasana*
17	Torca, La Francia	M1	*Myrcianthes leucoxyla*
18	Torca, La Francia	M1	*Vallea stipularis*
19	Torca, La Francia	M1	*Bejaria resinosa*
20	Torca, La Francia	M1	*Myrsine coriacea*
21	Torca, La Francia	M1	*Cytarexylum sulcatum*
22	Torca, La Francia	M1	*Macleania rupestris*
23	Torca, La Francia	M1	*Smilax tomentosa*
24	Torca, La Francia	M1	*Bejaria aestuans*
25	Torca, La Francia	M1	*Ilex kunthiana*
26	Torca, La Francia	M1	*Bidens pilosa*
27	Torca, La Francia	M1	*Bejaria resinosa*
28	Torca, La Francia	M1	*Oreopanax incisus*
29	Torca, La Francia	M1	*Daphnopsis caracasana*
30	Torca, La Francia	M1	*Daphnopsis caracasana*
31	Torca, La Francia	M1	*Myrcianthes leucoxyla*
32	Torca, La Francia	M1	*Daphnopsis caracasana*
33	Torca, La Francia	M1	*Daphnopsis caracasana*
34	Torca, La Francia	M1	*Xylosma spiculifera*
35	Torca, La Francia	M1	*Psycothrya boqueronensis*
36	Torca, La Francia	M1	*Miconia squamulosa*
37	Torca, La Francia	M1	*Psycothrya boqueronensis*
38	Torca, La Francia	M1	*Xylosma spiculifera*
39	Torca, La Francia	M1	*Myrsine coriacea*
40	Torca, La Francia	M1	*Daphnopsis caracasana*
41	Torca, La Francia	M1	*Daphnopsis caracasana*
42	Torca, La Francia	M2	*Alnus accuminata*
43	Torca, La Francia	M2	*Cavendishia bracteata*
44	Torca, La Francia	M2	*Vallea stipularis*
45	Torca, La Francia	M2	*Cavendishia bracteata*
46	Torca, La Francia	M2	*Cavendishia nitida*
47	Torca, La Francia	M2	*Vallea stipularis*
48	Torca, La Francia	M2	*Hesperomeles goudotiana*
49	Torca, La Francia	M2	*Diplostephium rosmarinifolius*
50	Torca, La Francia	M2	*Viburnum tryphyllum*
51	Torca, La Francia	M2	*Miconia squamulosa*
52	Torca, La Francia	M2	*Viburnum tinoides*
53	Torca, La Francia	M2	*Viburnum tinoides*
54	Torca, La Francia	M2	*Bejaria aestuans*
55	Torca, La Francia	M2	*Clusia multiflora*
56	Torca, La Francia	M2	*Viburnum tryphyllum*
57	Torca, La Francia	M2	*Daphnopsis caracasana*
58	Torca, La Francia	M2	*Daphnopsis caracasana*
59	Torca, La Francia	M2	*Daphnopsis caracasana*
60	Torca, La Francia	M2	*Xylosma spiculifera*
61	Torca, La Francia	M2	*Daphnopsis caracasana*
62	Torca, La Francia	M2	*Cavendishia bracteata*
63	Torca, La Francia	M2	*Myrcianthes leucoxyla*
64	Torca, La Francia	M2	*Gaiadendron punctatum*
65	Torca, La Francia	M2	*Oreopanax incisus*
66	Torca, La Francia	M2	*Oreopanax incisus*
67	Torca, La Francia	M1	*Xylosma spiculifera*
68	Torca, La Francia	M2	*Bejaria resinosa*
69	Torca, La Francia	M2	*Miconia squamulosa*
70	Torca, La Francia	M2	*Hesperomeles goudotiana*
71	Torca, La Francia	M2	*Myrsine pellucida*
72	Torca, La Francia	M2	*Viburnum tinoides*
73	Torca, La Francia	M2	*Myrsine guianensis*
74	Torca, La Francia	M2	*Aiouea dubia*
75	Torca, La Francia		*Alnus accuminata*
76	Torca, La Francia	M3	*Cavendishia bracteata*
77	Torca, La Francia	M3	*Gaiadendron punctatum*
78	Torca, Colegio	M4	*Gaiadendron punctatum*
79	Torca, Colegio	M4	*Peperomia rotundata*
80	Torca, Colegio	M4	*Frangula sphaerosperma*
81	Torca, Colegio	M4	*Cavendishia nitida*
82	Torca, Colegio	M4	*Palicourea angustifolia*
86	San Juan de Sumapaz, don Hernan	M8	*Hesperomeles ferruginea*
87	Torca colegio	M5	*Aiouea dubia*
88	Torca colegio	M5	*Phyllantus salvifolius*
89	Pasquilla los Encenillales	M2‐M6	*Gaiadendron punctatum*
90	Pasquilla los Encenillales	M2‐M6	*Bejaria resinosa*
91	Pasquilla los Encenillales	M2‐M6	*Myrsine dependens*
92	Pasquilla los Encenillales	M2‐M6	*Myconia ligustrina*
93	Pasquilla los Encenillales	M2‐M6	*Diplostephium rosmarinifolius*
94	Pasquilla los Encenillales	M2‐M6	*Vallea stipularis*
95	Pasquilla los Encenillales	M2‐M6	*Cavendishia bracteata*
96	Pasquilla los Encenillales	M2‐M6	*Hesperomeles goudotiana*
97	Pasquilla los Encenillales	M2‐M6	*Bucquetia glutinosa*
98	Pasquilla los Encenillales	M2‐M6	*Viburnum tryphyllum*
99	Pasquilla los Encenillales	M2‐M6	*Ageratina glyptophlebia*
100	Torca la francia	M1	*Peperomia glabella*
101	Torca la francia	M1	*Pleopeltis macrocarpa*
102	Torca la francia	M1	*Pleopeltis murora*
103	Torca la francia	M1	*Pleurothallis linguifera*
104	Pasquilla los Encenillales	M2	*Ponthieva similis*
105	Torca colegio	M4	*Chromolaena perglabra*
106	Torca colegio	M4	*Peperomia suratana*
107	Torca colegio	M4	*Epidendrum* sp.
108	Torca colegio	M4	*Serpocaulon eleutherophlebium*
109	Pasquilla los Encenillales	M2‐M6	*Hymenophyllum myriocarpum*
110	Pasquilla los Encenillales	M2‐M6	*Melpomene flabelliformis*
111	Pasquilla los Encenillales	M2‐M6	*Pecluma paradisiaca*
112	Pasquilla los Encenillales	M2‐M6	*Elaphpglossum gayanum*
113	Pasquilla los Encenillales	M2‐M6	*Hydrocotile tenerrima*
114	Pasquilla los Encenillales	M2‐M6	*Lycopodium clavatum*
115	Pasquilla los Encenillales	M2‐M6	*Bidens triplinervia*
116	Pasquilla los Encenillales	M2‐M6	*Passiflora adulterina*
117	Pasquilla los Encenillales	M2‐M6	*Malaxis crispifolia*
118	Torca colegio	M5	*Pilea parietaria*
119	Torca colegio	M5	*Thelypteris* sp.
120	Torca colegio	M5	*Pilea* sp.
121	Torca colegio	M5	*Gaultheria* sp.
122	Sumapaz san Juan	M7‐M7bis	*Ageratina glyptophlebia*
123	Sumapaz san Juan	M7‐M7bis	*Miconia eleanoides*
124	Sumapaz san Juan	M7‐M7bis	*Hesperomeles goudotiana*
125	Sumapaz san Juan	M7‐M7bis	*Hesperomeles ferruginea*
126	Sumapaz san Juan	M7‐M7bis	*Ilex* sp.
127	Sumapaz san Juan	M7‐M7bis	*Persea ferruginea*
128	Sumapaz san Juan	M7‐M7bis	*Vaccinium floribundum*
129	Sumapaz san Juan	M7‐M7bis	*Ageratina glyptophlebia*
130	Sumapaz san Juan	M7‐M7bis	*Ilex kunthiana*
131	Torca‐ conjunto floresta	R11‐R12‐R14	*Serpocaulon sessilifolium*
132	Torca‐ conjunto floresta	R11‐R12‐R14	*Epidendrum excisum*
133	Torca‐ conjunto floresta	R11‐R12‐R14	*Elleanthus purpureus*
134	Torca‐ conjunto floresta	R11‐R12‐R14	*Stelis cassidis*
135	Torca‐ conjunto floresta	R11‐R12‐R14	*Epidendrum brachyorodochilum*
136	Torca‐ conjunto floresta	R11‐R12‐R14	*Pleurothallis* sp.
137	Torca‐ conjunto floresta	R11‐R12‐R14	*Stelis* sp.
138	Torca‐ conjunto floresta	R11‐R12‐R14	*Frangula sphaerosperma*
139	Torca‐ conjunto floresta	R11‐R12‐R14	*Ocotea longifolia*
140	Tabio	R8‐R7	*Cardamine ovata*
141	Tabio	R8‐R7	*Valeriana clematis*
142	Tabio	R8‐R7	*Rhyncospora nervosa*
143	Tabio	R8‐R7	*Zeugites* sp.
144	Tabio	R8‐R7	*Asplenium praemorsum*
145	Tabio	R8‐R7	*Oxalis acetosella*
146	Tabio	R8‐R7	*Tradescantia* sp.
147	Tabio	R8‐R7	*Lantana* sp.
148	Tabio	R8‐R7	*Palicourea linearifolia*
149	Tabio	R8‐R7	*Piper bogotense*
150	Tabio	R8‐R7	*Daphnopsis caracasana*
151	Tabio	R8‐R7	*Peperomia angularis*
152	Tabio	R8‐R7	*Cuphea hyssopifolia*
153	Tabio	R8‐R7	*Peperomia galioides*
154	Tabio	R8‐R7	*Asteraceae* sp.
155	Tabio	R8‐R7	*Galianthe bogotensis*
156	Tabio	R8‐R7	*Anthoxanthum odoratum*
157	Tabio	R8‐R7	*Setaria italica*
158	Tabio	R8‐R7	*Serpocaulon laevigatum*
159	Tabio	R8‐R7	*Uncinia hamata*
160	Sumapaz san Juan	M8‐M8bis	*Ageratina tinifolia*
161	Sumapaz san Juan	M8‐M8bis	*Baccharis macrantha*
162	Sumapaz san Juan	M8‐M8bis	*Rhyncospora macrochaeta*
163	Sumapaz san Juan	M8‐M8bis	*Miconia resima*
164	Sumapaz san Juan	M8‐M8bis	*Carex pichinchensis*
165	Sumapaz san Juan	M8‐M8bis	*Rubus acantophilus*
166	Sumapaz san Juan	M8‐M8bis	*Bucquetia glutinosa*
167	Sumapaz san Juan	M8‐M8bis	*Escallonia myrtillioides*
168	Sumapaz san Juan	M8‐M8bis	*Hymenophyllum myriocarpum*
169	Sumapaz san Juan	M8‐M8bis	*Podocarpus oleifolia*
170	Sumapaz san Juan	M8‐M8bis	*Ilex* sp.
171	Sumapaz san Juan	M8‐M8bis	*Miconia eleanoides*
172	Sumapaz san Juan	M8‐M8bis	*Calceolaria microbefaria*
173	Torca‐ conjunto floresta	R13	*Conyza canadensis*
174	Torca‐ conjunto floresta	R13	*Arrenatherum elatius*
175	Torca‐ conjunto floresta	R13	*Peperomia accuminata*
176	Torca‐ conjunto floresta	R13	*Fuchsia boliviana*
177	Torca‐ conjunto floresta	R13	*Serpocaulon laevigatum*
178	Torca‐ conjunto floresta	R13	*Criotonopsis bogotana*
179	Torca‐ conjunto floresta	R13	*Miconia resima*
180	Torca‐ conjunto floresta	R13	*Fragaria vesca*
182	Torca‐ conjunto floresta	R13	*Gamochaete americana*
183	Tabio	R9‐R10	*Pleopeltis macrocarpa*
184	Tabio	R9‐R10	*Pleopeltis murora*
185	Tabio	R9‐R10	*Peperomia cf spatulata*
186	Tabio	R9‐R10	*Botrichium virginianum*
187	Tabio	R9‐R10	*Peperomia* sp.
188	Tabio	R9‐R10	*Asplenium sp*.
189	Tabio	R9‐R10	*Oxalis cf macrocarpa*
190	Tabio	R9‐R10	*Stelis* sp.
191	Tabio	R9‐R10	*Miconia squamulosa*
192	Tabio	R9‐R10	*Cestrum buxifolium*
193	Tabio	R9‐R10	*Alonsoa meridionalis*
194	Tabio	R9‐R10	*Pilea lindeniana*
195	Tabio	R9‐R10	*Epidendrum scutella*
196	Tabio	R9‐R10	*Phyllantus salvifolius*
197	Tabio	R9‐R10	*Galium* sp.
198	Tabio	R9‐R10	*Palicourea linearifolia*
199	Tabio	R9‐R10	*Uncinia hamata*
200	Tabio	R9‐R10	*Xylosma spiculifera*
201	Tabio	R9‐R10	*Clematis*
202	Tabio	R9‐R10	*Peperomia suratana*
203	Tabio	R9‐R10	*Rhyncospora macrochaeta*
204	Tabio	R9‐R10	*Cardamine ovata*
205	Tabio	R9‐R10	*Ponthieva similis*
206	Tabio	R9‐R10	*Ageratina* sp.
207	Encenillo	R3‐R5	*Stelis* sp.
208	Encenillo	R3‐R5	*Lepanthes gargantua*
209	Encenillo	R3‐R5	*Nertera granadensis*
210	Encenillo	R3‐R5	*Pleurothallis lindenii*
211	Encenillo	R3‐R5	*Huperzia* sp.
212	Encenillo	R3‐R5	*Stelis* sp.
213	Encenillo	R3‐R5	*Sphyrospermum buxifolium*
214	Encenillo	R3‐R5	*Peperomia arthurii*
215	Pasquilla Finca Alveiro	M9bis‐M10	*Smallanthus pyramidalis*
216	Pasquilla Finca Alveiro	M9bis‐M10	*Elleanthus aurantiacus*
217	Pasquilla Finca Alveiro	M9bis‐M10	*Rhyncospora* sp.
218	Pasquilla Finca Alveiro	M9bis‐M10	*Peperomia microphylla*
219	Pasquilla Finca Alveiro	M9bis‐M10	*Gaultheria anastomosans*
220	Pasquilla Finca Alveiro	M9bis‐M10	*Epidendrum caesaris*
221	Pasquilla Finca Alveiro	M9bis‐M10	*Elaphoglossum lindenii*
222	Pasquilla Finca Alveiro	M9bis‐M10	*Apiaceae* sp.
223	Pasquilla Finca Alveiro	M9bis‐M10	*Serpocaulon lasiopus*
224	Pasquilla Finca Alveiro	M9bis‐M10	*Melastomataceae*
225	Pasquilla Finca Alveiro	M9bis‐M10	*Digitalis purpurea*
226	Pasquilla Finca Alveiro	M9bis‐M10	*Oxalis cf spiralis*
227	Pasquilla Finca Alveiro	M9bis‐M10	*Berberis rigidifolia*
228	Pasquilla Finca Alveiro	M9bis‐M10	*Bidens* sp.
229	Pasquilla Finca Alveiro	M9bis‐M10	*Elaphoglossum gayanum*
230	Pasquilla Finca Alveiro	M9bis‐M10	*Baccharis bogotensis*
231	Pasquilla Finca Alveiro	M9bis‐M10	*Ageratina asclepiadea*
232	Pasquilla Finca Alveiro	M9bis‐M10	*Weinmannia tomentosa*
233	Pasquilla Finca Alveiro	M9bis‐M10	*Hypochaeris radicata*
234	Pasquilla Finca Alveiro	M9bis‐M10	*Peperomia* sp.
235	Pasquilla Finca Alveiro	M9bis‐M10	*Cytarexylum sulcatum*
236	Pasquilla Finca Alveiro	M9bis‐M10	*Morella parvifolia*
237	Pasquilla Finca Alveiro	M9bis‐M10	*Vallea stipularis*
238	Pasquilla Finca Alveiro	M9bis‐M10	*Bidens* sp.
239	Pasquilla Finca Alveiro	M9bis‐M10	*Galium* sp.
240	Pasquilla Finca Alveiro	M9bis‐M10	*Hypericum juniperinum*
241	Pasquilla Finca Alveiro	M9bis‐M10	*Vaccinium floribundum*
242	Torca‐Portal de Fusca	R17‐R18	*Campyloneuron latum*
243	Torca‐Portal de Fusca	R17‐R18	*Athyrium flix‐femina*
244	Torca‐Portal de Fusca	R17‐R18	*Clethra fimbriata*
245	Torca‐Portal de Fusca	R17‐R18	*Hieracium avilae*
246	Torca‐Portal de Fusca	R17‐R18	*Cranichis ciliata*
247	Torca‐Portal de Fusca	R17‐R18	*Nyphogeton* sp.
248	Torca‐Portal de Fusca	R17‐R18	*Jungia ferruginea*
249	Torca‐Portal de Fusca	R17‐R18	*Frangula goudotiana*
250	Torca‐Portal de Fusca	R17‐R18	*Pilea sp foglie serrate*
251	Torca‐Portal de Fusca	R17‐R18	*Syphocampilus columnae*
252	Torca‐Portal de Fusca	R17‐R18	*Pilea lindeniana*
253	Torca‐Portal de Fusca	R17‐R18	*Uncinia hamata*
254	Torca‐Portal de Fusca	R17‐R18	*Asplenium monantes*
255	Torca‐Portal de Fusca	R17‐R18	*Peperomia suratana*
256	Torca‐Portal de Fusca	R17‐R18	*Carex* sp.
257	Torca‐Portal de Fusca	R17‐R18	*Eriosorus flexuosus*
258	Torca‐Portal de Fusca	R17‐R18	*Asplenium cladolepton*
265	Encenillo	R15‐R16	*Fernandezia sanguinea*
266	Encenillo	R15‐R16	*Macrocarpaea glabra*
267	Encenillo	R15‐R16	*Frangula goudotiana*
268	Encenillo	R15‐R16	*Stelis* sp.
269	Encenillo	R15‐R16	*Diphasiastrum thyoides*
270	Encenillo	R15‐R16	*Ponthieva villosa*
271	Encenillo	R15‐R16	*Stelis* sp.
272	Encenillo	R15‐R16	*Stelis* sp.
273	Tabio	R20	*Maxillaria graminifolia*
274	Tabio	R19	*Phenax rugosus*
275	Tabio	R19	*Pleurothallis* sp.
277	Tabio	R19	*Pecluma divaricata*
278	Tabio	R19	*Pleopeltis murora*
279	Tabio	R19	*Cystopteris fragilis*
280	Tabio	R19	*Adiantum andicola*
281	Tabio	R19	*Asplenium radicans*

## APPENDIX A4

### List of retrievable aerial pictures

**Table:**

Quadrant	Year	Folder number	Flight number	Picture number	Corresponding plot
K‐11	1962	s‐222336A	c‐1063	1800	Torca
K‐11	1940	s‐501	a‐136	157;156	Torca
K‐11	1940	s‐777	c‐16	387;385;383;381	Torca
K‐11	1962	22214B	c‐1058	1165;1164	Torca
K‐11	1978	s‐4542	r‐750	153;152;151	Torca
K‐11	1986	digitalized	c‐2265	9	Torca
K‐11	1993	digitalized	c‐2523	217	Torca
K‐11	2000	digitalized	r‐1212	129	Torca
K‐11	2004	digitalized	c‐2717	259	Torca
K‐10	1940	s‐913	c‐71	704;703	Tabio
K‐10	1957	s‐239	m‐127	1863	Tabio
K‐10	1961	s‐22340	c‐1082	2478	Tabio
K‐10	1993	s‐36949	c‐2521	82	Tabio
K‐10	1998	s‐37851	c‐2636	225	Tabio
K‐10	2007	s‐40778	c‐2800	83	Tabio
L‐10	1948	s‐2159	c‐502	281	Sumapaz
L‐10	1951	s‐2715	c‐606	290	Sumapaz
L‐10	1961	s‐2282	R‐487	46	Sumapaz
L‐10	1963	s‐1136	M‐1266	26084	Sumapaz
L‐10	1987	s‐34455	c‐2323	220	Sumapaz
L‐10	1996	s‐37521B	c‐2584	120	Sumapaz
L‐10	1941	s‐668	a‐232	72;70	Pasquilla
L‐10	1961	s‐895	m‐1142	18941;18940	Pasquilla
L‐10	1977	s‐29014	?	81:86	Pasquilla
L‐10	1981	s‐30657	c‐1985	94	Pasquilla
L‐10	1993	s‐36950	c‐2521	129	Pasquilla
L‐10	2007	s‐40802	c‐2803	170	Pasquilla
K‐11	1940	s‐530	a‐148	152	Guatavita
K‐11	1958	s‐21249	c‐859	531	Guatavita
K‐11	1962	s‐222236B	c‐1063	1812	Guatavita
K‐11	1997	digitalized	c‐2611	12	Guatavita
K‐11	2007	digitalized	c‐2799	63	Guatavita
K‐11	?	digitalized	c‐2471	54	Guatavita
K‐11	?	digitalized	c‐2673	667	Guatavita
K‐11	1940	s‐501	a‐136	164	Guasca
K‐11	1955	s‐148	m‐46	4475	Guasca
K‐11	1958	s‐21265	c‐860	174	Guasca
K‐11	1963	s‐22194A	c‐1055	425	Guasca
K‐11	1978	s‐29190	c‐1808	13	Guasca
K‐11	1985	digitalized	c‐2183	39	Guasca
K‐11	1993	digitalized	c‐2523	144	Guasca
K‐11	2007	digitalized	c‐2799	56	Guasca
K‐11	2010	digitalized	22803002012010	500	Guasca

## APPENDIX A5

### Indicator species analysis values (IVI) for tree and understory layer

TREES

**Table:**

*Species*	Cluster	Value (IV)	Mean	*SD*	*p* ^*^
*Miconia elaeoides*	1	92	22.8	8.87	.0002
*Myrcianthes leucoxyla*	1	63.4	26.7	11.24	.0122
*Viburnum triphyllum*	1	52	26.2	8.07	.0062
*Vallea stipularis*	1	32.8	25.8	7.42	.1664
*Psychotria boqueronensis*	1	30	18.7	10.34	.144
*Aiouea dubia*	1	20	18.7	9.16	.4105
*Duranta mutisii*	1	20	19.1	9.58	.5653
*Frangula sphaerosperma*	1	20	18.6	8.89	.2769
*Lippia hirsuta*	1	20	18.7	8.82	.4151
*Maytenus laxiflora*	1	20	19.2	10	.4747
*Symplocos theiformis*	1	18.5	20	10.47	.3391
*Abatia parviflora*	1	10	18.7	7.84	1
*Barnadesia spinosa*	1	10	18.7	7.84	1
*Carica sp*	1	10	18.7	7.84	1
*Melastomataceae NA*	1	10	18.7	7.84	1
*Myrsine pellucida*	1	10	18.9	8	1
*Ocotea heterocroma*	1	10	18.8	7.9	1
*Pentacalia NA*	1	10	18.7	8	1
*Phyllanthus salviifolius*	1	10	18.8	7.9	1
*Sessea corymbosa*	1	10	18.8	7.98	1
*Monticalia pulchella*	2	80.1	23.3	11.3	.0002
*Macleania rupestris*	2	76.6	27.8	10.25	.0002
*Ilex kunthiana*	2	65.9	29.5	9.65	.001
*Myrcianthes ropaloides*	2	50	18.6	9.01	.045
*Ageratina asclepiadea*	2	45.1	25	13.52	.0792
*Viburnum tinoides*	2	34.8	19.5	10.85	.1012
*Diplostephium ochraceum*	2	30.8	18	10.22	.1236
*Tibouchina grossa*	2	25	18.8	7.93	.4757
*Gaultheria anastomosans*	3	100	19.3	10.97	.0004
*Ageratina glyptophlebia*	3	90.9	24.9	12.84	.0006
*Bucquetia glutinosa*	3	77.2	24.4	12.2	.0028
*Ageratina boyacensis*	3	75	20	10.42	.0032
*Berberis glauca*	3	75	20	11.34	.0038
*Vaccinium floribundum*	3	75	18.2	10.33	.0038
*Myrsine dependens*	3	67.5	23.3	11.28	.0056
*Ageratina tinifolia*	3	50	19.6	10.34	.0462
*Blechnum schomburgkii*	3	50	20.7	10.27	.0468
*Hesperomeles ferruginea*	3	50	19.3	9.97	.0462
*Ilex sp1*	3	50	18.9	9.17	.0468
*Persea ferruginea*	3	50	20.1	10.38	.0468
*Polylepis quadrijuga*	3	50	20.8	10.26	.0468
*Weinmannia fagaroides*	3	49.5	20.6	10.28	.046
*Hesperomeles goudotiana*	3	46	28.2	10.38	.0672
*Miconia ligustrina*	3	44.9	22.7	12.58	.076
*Clethra fagifolia*	3	25	18.7	7.88	.4699
*Escallonia myrtilloides*	3	25	18.8	7.92	.4701
*Hesperomeles obtusifolia*	3	25	18.9	7.98	.4753
*Podocarpus oleifolia*	3	25	18.8	7.92	.4701
*Cestrum buxifolium*	3	20.3	19.2	10.02	.4803
*Myrsine coriacea*	4	71.2	23.8	8.76	.0002
*Clusia multiflora*	4	67.7	21.9	11.04	.0038
*Drimys granadensis*	4	61.2	22.8	11.49	.0076
*Weinmannia tomentosa*	4	45.8	25.4	8.02	.0214
*Hediosmum sp*	4	42.9	20.3	11.21	.0846
*Bejaria resinosa*	4	41.7	23.6	9.28	.0532
*Cavendishia nitida*	4	31.5	22.2	11.12	.1824
*Macrocarpaea glabra*	4	28.6	19	9.56	.123
*Ocotea calophylla*	4	28.6	18.8	8.78	.0838
*Palicourea demissa*	4	28.6	18.5	8.84	.085
*Myrsine latifolia*	4	27.5	22.7	11.48	.2296
*Frangula goudotiana*	4	24.6	20.5	9.86	.2454
*Critoniopsis bogotana*	4	16.5	20.2	10.33	.5909
*Clethra lanata*	4	14.3	18.7	7.99	.6699
*Cybianthus iteoides*	4	14.3	18.8	7.94	.6927
*Aiouea sp1*	4	11.4	19.4	10.29	.7518
*Cavendishia bracteata*	5	80.5	29	10.32	.0004
*Diplostephium rosmarinifolius*	5	78.5	29.7	13.98	.0054
*Gaiadendron punctatum*	5	75.2	22.1	10.95	.0018
*Ulex europaeus*	5	66.7	19.7	10.14	.0064
*Alnus acuminata*	5	54	19.1	10.64	.0148
*Clethra fimbriata*	5	43.9	20.9	11.39	.0528
*Oreopanax bogotensis*	5	36.4	20.9	11.38	.1054
*Ageratina fastigiata*	5	33.3	18.8	7.99	.096
*Baccharis prunifolia*	5	33.3	18.8	7.99	.096
*Varronia cylindrostachya*	6	73.1	20.9	11.42	.002
*Myrsine guianensis*	6	60	32.5	13.06	.0498
*Oreopanax incisus*	6	51.8	24.4	9.37	.0186
*Daphnopsis caracasana*	6	47	22.2	10.07	.023
*Miconia squamulosa*	6	46	28.8	11.19	.085
*Piper bogotense*	6	45.2	22.7	12.22	.0546
*Xylosma spiculifera*	6	40.1	23	10.16	.0666
*Palicourea lineariflora*	6	36.8	23.7	11.49	.1302
*Baccharis macrantha*	6	25	18.6	7.91	.4609
*Bocconia frutescens*	6	25	18.7	7.88	.4653
*Cestrum sp*	6	25	18.7	7.88	.4653
*Solanum cornipholium*	6	25	18.7	7.92	.4663
*Valeriana clematitis*	6	25	18.6	7.91	.4609
*Verbesina arborea*	6	22.8	19.3	10.18	.3721
*Prunus buxifolia*	6	22.6	19.2	10.09	.3765
*Citharexylum sulcatum*	6	21.1	22	11.9	.3553
*Cedrela montana*	6	19.8	19.5	10.43	.3109
*Escallonia paniculata*	6	18.9	18.6	8.9	.4337
*Myrica parvifolia*	6	18	21.6	11.77	.4799
*Palicourea angustifolia*	6	16	21.8	11.46	.6393
*Croton bogotanus*	6	13.5	19.1	10.31	.7337
*Myrica pubescens*	6	13	19.5	10.8	.7037

UNDERSTORY

**Table:**

Species	Group	Value (IV)	Mean	*SD*	*p* ^*^
*Oreopanax incisus*	1	78	25.1	11.18	.0002
*Passiflora bogotensis*	1	66.2	24.2	11.73	.0118
*Serpocaulon levigatum*	1	59.7	24.3	13.14	.0304
*Piper bogotense*	1	54.5	20.9	11.46	.0212
*Oligactis sessiliflora*	1	54.2	26.8	14.52	.033
*Blechnum occidentale*	1	53.8	22	11.93	.0336
*Smilax tomentosa*	1	50.5	31.4	14.34	.0798
*Cedrela montana*	1	44	24.4	14.07	.1132
*Miconia squamulosa*	1	41.7	28.8	12.23	.1228
*Frangula sphaerosperma*	1	39.8	28.8	12.86	.1474
*Uncinia hamata*	1	39.4	26.2	13.1	.1168
*Peperomia suratana*	1	39.2	27.4	11.95	.1384
*Xylosma spiculifera*	1	37.3	21.7	11.9	.0738
*Maxillaria graminifolia*	1	36.4	22.4	13.36	.1552
*Pleurothallis linguifera*	1	36.4	19.3	11.93	.0886
*Anthoxanthum odoratum*	1	34.9	25.2	12.98	.1844
*Smilax sp1*	1	33.3	26.1	11.97	.2146
*Daphnopsis caracasana*	1	29.5	25.6	13.12	.2757
*Barnadesia spinosa*	1	28.9	20.9	12.45	.1968
*Palicourea angustifolia*	1	28.8	25	11.16	.2621
*Palicourea lineariflora*	1	28.4	20	11.31	.1848
*Critoniopsis bogotana*	1	27.3	19	12.4	.2354
*Pilea lindeniana*	1	27.3	19.6	12.43	.1992
*Valeriana clematitis*	1	25.1	31.5	10.51	.6753
*Stelis sp1*	1	23.4	22.4	11.79	.3619
*Frangula sp1*	1	19.3	22.1	12.16	.4903
*Ageratina gracilis*	1	18.2	17.9	11.18	.4121
*Boehmeria cylindrica*	1	18.2	17.1	11.63	.3429
*Botrychium virginianum*	1	18.2	17.2	11.99	.3413
*Chromolaena perglabra*	1	18.2	17.9	10.91	.4119
*Chromolaena scabra*	1	18.2	17.4	11.23	.3811
*Chromolaena sp1*	1	18.2	16.8	11.37	.3353
*Clematis dioica*	1	18.2	17.1	11.63	.3359
*Clematis haenkeana*	1	18.2	17	11.49	.3415
*Conyza canadensis*	1	18.2	17	11.2	.3287
*Maxillaria sp1*	1	18.2	17.8	11.17	.4121
*Pecluma divaricata*	1	18.2	17	11.76	.3359
*Peperomia emarginulata*	1	18.2	17	11.6	.3333
*Pilea alsinifolia*	1	18.2	17.6	10.9	.4031
*Solanum cornifolium*	1	18.2	17.8	11.04	.4117
*Stelis pulchella*	1	18.2	17.1	11.69	.3465
*Tillandsia complanata*	1	18.2	17	11.74	.3465
*Chromolaena bullata*	1	14.9	20	12.39	.5255
*Varronia cylindrostachya*	1	14.9	19.6	12.31	.5537
*Bomarea sp1*	1	14.6	25.3	12.66	.841
*Capsella bursapastoris*	1	14.6	19.6	12.22	.5441
*Miconia theizans*	1	14.3	19.5	12.12	.5299
*Pleopeltis macrocarpa*	1	13.6	20.7	12.09	.6725
*Epidendrum moritzii*	1	12.3	17.9	11.19	.5925
*Citharexylum sulcatum*	1	9.6	17.9	11.79	.6985
*Adiantum andicola*	1	9.1	15.7	9.87	1
*Alansmia sp1*	1	9.1	15.7	9.75	1
*Alonsoa meridionalis*	1	9.1	15.8	9.95	1
*Anchietea frangulifolia*	1	9.1	15.9	10.04	1
*Anthurium caramantae*	1	9.1	15.7	9.75	1
*Arrhenatherum elatius*	1	9.1	15.6	9.53	1
*Asplenium cladolepton*	1	9.1	15.5	9.69	1
*Asplenium praemorsum*	1	9.1	15.5	9.48	1
*Asplundianthus densus*	1	9.1	15.6	9.71	1
*Baccharis latifolia*	1	9.1	15.6	9.53	1
*Boehmeria sp1*	1	9.1	15.7	9.75	1
*Campyloneurum latum*	1	9.1	15.5	9.69	1
*Carex sp1*	1	9.1	15.5	9.69	1
*Castilleja fissifolia*	1	9.1	15.5	9.59	1
*Chromolaena leivensis*	1	9.1	15.6	9.71	1
*Croton bogotanus*	1	9.1	15.8	9.95	1
*Cuphea hyssopifolia*	1	9.1	15.5	9.48	1
*Cyperus sp1*	1	9.1	15.6	9.53	1
*Cystopteris fragilis*	1	9.1	15.7	9.87	1
*Dryopteris sp1*	1	9.1	15.5	9.69	1
*Duranta mutisii*	1	9.1	15.4	9.46	1
*Epidendrum sp3*	1	9.1	15.9	10.04	1
*Epidendrum sp4*	1	9.1	15.6	9.64	1
*Fragaria vesca*	1	9.1	15.6	9.53	1
*Fuchsia boliviana*	1	9.1	15.6	9.53	1
*Fuchsia paniculata*	1	9.1	15.5	9.69	1
*Galianthe bogotensis*	1	9.1	15.5	9.48	1
*Gamochaeta americana*	1	9.1	15.6	9.53	1
*Heppiella ulmifolia*	1	9.1	15.5	9.69	1
*Lantana rugosa*	1	9.1	15.5	9.48	1
*Lepidaploa canescens*	1	9.1	15.5	9.59	1
*Malaxis crispifolia*	1	9.1	15.7	9.75	1
*Oxalis acetosella*	1	9.1	15.5	9.48	1
*Panicum sp1*	1	9.1	15.5	9.48	1
*Passiflora sp1*	1	9.1	15.8	9.95	1
*Passiflora tripartita*	1	9.1	15.5	9.69	1
*Peperomia glabella*	1	9.1	15.6	9.71	1
*Phenax rugosus*	1	9.1	15.7	9.87	1
*Phyllanthus salviifolius*	1	9.1	15.7	9.75	1
*Physalis peruviana*	1	9.1	15.4	9.46	1
*Pilea goudotiana*	1	9.1	15.5	9.69	1
*Piper marginatum*	1	9.1	15.7	9.87	1
*Ponthieva similis*	1	9.1	15.5	9.59	1
*Pteris muricata*	1	9.1	15.7	9.87	1
*Rubus macrocarpus*	1	9.1	15.7	9.75	1
*Rubus sp1*	1	9.1	15.6	9.53	1
*Salvia sp1*	1	9.1	15.8	9.95	1
*Setaria italica*	1	9.1	15.5	9.48	1
*Solanum caripense*	1	9.1	15.4	9.46	1
*Solanum pseudocapsicum*	1	9.1	15.7	9.87	1
*Solanum sp1*	1	9.1	15.5	9.69	1
*Solanum sp2*	1	9.1	15.7	9.87	1
*Stelis sp2*	1	9.1	15.5	9.59	1
*Stenorrhynchos speciosum*	1	9.1	15.6	9.53	1
*Styrax sp1*	1	9.1	15.6	9.64	1
*Thelipteris sp1*	1	9.1	15.7	9.75	1
*Tigridia pavonia*	1	9.1	15.5	9.48	1
*Tillandsia sp5*	1	9.1	15.5	9.48	1
*Tradescantia sp1*	1	9.1	15.5	9.48	1
*Tradescantia sp2*	1	9.1	15.8	9.95	1
*Vasquezia anemonifolia*	1	9.1	15.5	9.48	1
*Cranichis ciliata*	1	8.2	17.6	11.02	.8812
*Miconia resima*	1	8.2	17.8	10.93	1
*Prunus sp1*	1	7.9	17.5	11.11	.9398
*Viburnum tinoides*	1	7.7	22.2	12.39	.975
*Berberis goudotii*	1	6.7	16.9	11.22	.937
*Achyrocline satureioides*	1	6.6	17.5	11.21	1
*Stelis sp3*	1	6	17	11.44	1
*Hypochaeris radicata*	1	5.8	17	11.38	1
*Asplenium radicans*	1	5.7	19.1	12.02	1
*Elaphoglossum lingua*	2	71.5	23.8	11.05	.001
*Chusquea scandens*	2	45.4	24.4	12.15	.0634
*Prunus buxifolia*	2	37	20	12.15	.0862
*Elaphoglossum cuspidatum*	2	36	24.7	12.78	.1622
*Frangula goudotiana*	2	34.8	24.2	13.27	.1654
*Galium hypocarpium*	2	30.6	28.2	12.45	.3527
*Tillandsia sp1*	2	27.3	22	12.54	.2665
*Clusia multiflora*	2	25	16.8	11.13	.2272
*Digitalis purpurea*	2	25	17.9	11.07	.173
*Diplostephium rosmarinifolium*	2	25	17.1	11.48	.2316
*Elleanthus aurantiacus*	2	25	17	11.37	.207
*Elleanthus purpureus*	2	25	16.7	11.15	.2196
*Hedyosmum racemosum*	2	25	17	11.58	.2244
*Ocotea longifolia*	2	25	17.2	11.48	.2078
*Serpocaulon lasiopus*	2	25	18	11.08	.173
*Smilax floribunda*	2	25	17.3	10.56	.1638
*Tillandsia sp3*	2	25	17.8	10.55	.1588
*Ocotea heterochroma*	2	23	18.7	12.21	.2875
*Rhyncospora sp1*	2	21.1	19.4	12.18	.3229
*Ilex kunthiana*	2	20.6	23.7	12.46	.4899
*Hieracium avilae*	2	17.7	20.7	12.56	.4343
*Blechnum cordatum*	2	16.8	18.3	11.71	.3985
*Munnozia senecionidis*	2	14.1	19.3	11.34	.6187
*Acaena cylindristachya*	2	12.5	15.6	9.57	.6645
*Athyrium dombeyi*	2	12.5	15.6	9.57	.6645
*Athyrium filixfemina*	2	12.5	15.6	9.83	.6439
*Baccharis lehmannii*	2	12.5	15.4	9.35	.6543
*Bejaria resinosa*	2	12.5	15.4	9.35	.6543
*Calamagrostis effusa*	2	12.5	15.6	9.57	.6645
*Chaetolepis lindeniana*	2	12.5	15.6	9.57	.6645
*Elaphoglossum lindenii*	2	12.5	15.6	9.79	.6505
*Elleanthus sp1*	2	12.5	15.4	9.35	.6543
*Epidendrum caesaris*	2	12.5	15.6	9.79	.6505
*Epidendrum cylindraceum*	2	12.5	15.6	9.57	.6561
*Epidendrum excisum*	2	12.5	15.5	9.67	.6523
*Eriosorus flexuosus*	2	12.5	15.6	9.83	.6439
*Faramea sp1*	2	12.5	15.6	9.83	.6439
*Fernandezia crystallina*	2	12.5	15.4	9.35	.6543
*Fernandezia sanguinea*	2	12.5	15.4	9.35	.6543
*Hypericum juniperinum*	2	12.5	15.6	9.57	.6645
*Lycopodium jussiaei*	2	12.5	15.5	9.47	.6583
*Macrocarpaea glabra*	2	12.5	15.4	9.35	.6543
*Myrsine sp1*	2	12.5	15.6	9.83	.6439
*Nyphogeton sp1*	2	12.5	15.6	9.79	.6505
*Paspalum bonplandianum*	2	12.5	15.6	9.57	.6645
*Ponthieva villosa*	2	12.5	15.4	9.35	.6543
*Sauvagesia erecta*	2	12.5	15.6	9.79	.6505
*Serpocaulon sessilifolium*	2	12.5	15.5	9.67	.6523
*Smilax sp2*	2	12.5	15.5	9.67	.6523
*Stelis argentata*	2	12.5	15.4	9.35	.6543
*Stelis galeata*	2	12.5	15.4	9.35	.6543
*Stelis pusilla*	2	12.5	15.4	9.35	.6543
*Stelis sp4*	2	12.5	15.4	9.35	.6543
*Stelis sp5*	2	12.5	15.4	9.35	.6543
*Stelis sp6*	2	12.5	15.4	9.35	.6543
*Oxalis spiralis*	2	11.4	17.7	11.02	.7798
*Oxalis corniculata*	2	11.2	17.2	11.32	.6857
*Peperomia microphylla*	2	11.1	17.9	11.26	.759
*Sphyrospermum buxifolium*	2	9.9	17.1	11.09	.7818
*Monochaetum bonplandii*	2	9.3	17.4	11.21	.7926
*Jungia ferruginea*	2	9.1	16.9	11.65	.7906
*Tibouchina grossa*	2	7.6	18.9	12.37	.9214
*Monnina aestuans*	3	100	17.6	11.34	.003
*Peperomia rotundata*	3	90.3	18.6	12.32	.0044
*Vaccinium floribundum*	3	87.1	23.5	13.73	.0112
*Nertera granadensis*	3	85.4	32	14.96	.0206
*Oreopanax bogotensis*	3	80.7	23	13.12	.0094
*Serpocaulon eleutherophlebium*	3	71.4	21.2	11.47	.0106
*Ageratina boyacensis*	3	50	15.7	9.76	.0638
*Arracacia sp1*	3	50	15.7	9.76	.0638
*Bomarea multiflora*	3	50	15.7	9.76	.0638
*Campyloneurum angustifolium*	3	50	15.7	9.76	.0638
*Equisetum bogotense*	3	50	15.7	9.76	.0638
*Fuchsia magellanica*	3	50	15.7	9.76	.0638
*Geranium holosericeum*	3	50	15.7	9.76	.0638
*Habenaria sp1*	3	50	15.7	9.76	.0638
*Hydrocotyle bonplandii*	3	50	15.7	9.76	.0638
*Miconia elaeoides*	3	50	15.7	9.76	.0638
*Oxalis medicaginea*	3	50	15.7	9.76	.0638
*Peperomia hartwegiana*	3	50	15.7	9.76	.0638
*Pleopeltis rudis*	3	50	15.7	9.76	.0638
*Rubus choachiensis*	3	50	15.7	9.76	.0638
*Serpocaulon murorum*	3	50	15.7	9.76	.0638
*Thelipteris sp2*	3	50	15.7	9.76	.0638
*Asplenium monanthes*	3	47.6	17.2	11.02	.028
*Oreopanax mutisianus*	3	46.9	17.2	11.46	.036
*Hydrocotyle tenerrima*	3	46.4	17.2	11.65	.0438
*Tillandsia sp2*	3	46.1	19.3	12.32	.0446
*Epidendrum scutella*	3	42.9	18.5	11.99	.0306
*Melpomene moniliformis*	3	38.1	18.1	11.71	.0656
*Galium ascendens*	3	38	18.6	12.36	.1008
*Bucquetia glutinosa*	3	37.8	20.8	12.71	.0884
*Berberis glauca*	3	36	18.3	12.18	.1064
*Passiflora adulterina*	3	31.9	18.2	11.81	.1366
*Siphocampylus brevicalyx*	3	30.3	18.4	11.67	.172
*Diphasiastrum thyoides*	3	29.1	19.7	11.97	.1978
*Elaphoglossum engelii*	3	27.7	19.4	11.92	.208
*Matelea mutisiana*	3	26.8	17.2	11.49	.1672
*Pentacalia pulchella*	3	24.4	20.1	12.42	.2801
*Pleopeltis sp1*	3	24.1	17.2	11.14	.1864
*Symplocos theifolia*	3	24.1	18.1	11.74	.3155
*Hesperomeles goudotiana*	3	22.3	26.9	14.08	.5485
*Clethra fimbriata*	3	21.8	17.2	11.1	.204
*Elaphoglossum gayanum*	3	16	19.7	12.19	.4997
*Orthrosanthus chimboracensis*	3	15.6	18.3	11.72	.4273
*Lycopodium clavatum*	3	14.5	20.6	12.29	.6869
*Ageratina glyptophlebia*	3	13.2	21.2	12.74	.6993
*Pleopeltis murora*	3	9.4	21	12.74	.9032
*Peperomia galioides*	3	8.7	21.4	12.8	.925
*Greigia stenolepis*	4	99.9	21.2	11.38	.0002
*Rubus acanthophyllos*	4	67.7	20.8	11.93	.0128
*Drimys granadensis*	4	54.7	22.2	11.9	.033
*Scyphostelma tenella*	4	54.1	21.7	12.68	.0102
*Myrsine dependens*	4	47.6	19.6	11.93	.019
*Elaphoglossum latifolium*	4	45.8	20.9	12.56	.0334
*Melpomene flabelliformis*	4	41	21.2	12.44	.0656
*Blechnum schomburgkii*	4	40	17.1	11.47	.086
*Hesperomeles obtusifolia*	4	40	17.8	10.93	.0838
*Huperzia hippuridea*	4	39.6	19.6	12.08	.086
*Luzula gigantea*	4	37.6	18.9	12.07	.0896
*Hymenophyllum myriocarpum*	4	36.8	21.1	12.25	.0692
*Persea ferruginea*	4	35.9	19.9	12.33	.1052
*Agrostis perennans*	4	34.7	21	12.68	.151
*Diplostephium ochraceum*	4	30.1	19.4	11.37	.1748
*Cestrum buxifolium*	4	25.8	24.9	13.12	.3467
*Elleanthus maculatus*	4	24.6	18.3	12.1	.2525
*Piper artanthe*	4	21.1	24.9	12.72	.5047
*Blechnum loxense*	4	20	15.7	9.62	.2234
*Calceolaria microbefaria*	4	20	15.9	10.1	.2212
*Carex pichinchensis*	4	20	15.9	10.1	.2212
*Diplostephium floribundum*	4	20	15.4	9.48	.206
*Elaphoglossum minutum*	4	20	15.7	9.62	.2234
*Escallonia myrtilloides*	4	20	15.4	9.48	.206
*Espeletiopsis corymbosa*	4	20	15.7	9.62	.2234
*Geissanthus andinus*	4	20	15.4	9.48	.206
*Hydrocotyle gunnerifolia*	4	20	15.8	10.05	.2196
*Ilex sp1*	4	20	15.4	9.48	.206
*Lepanthes gargantua*	4	20	15.5	9.59	.2158
*Miconia latifolia*	4	20	15.7	9.62	.2234
*Monnina fastigiata*	4	20	15.7	9.62	.2234
*Pecluma sp1*	4	20	15.8	10.05	.2196
*Pentacalia nitida*	4	20	15.4	9.48	.206
*Peperomia alibacophylla*	4	20	15.4	9.48	.206
*Pernettya gaultheria*	4	20	15.4	9.48	.206
*Pilea sp1*	4	20	15.4	9.48	.206
*Plagiogyria pectinata*	4	20	15.7	9.62	.2234
*Pleurothallis lindenii*	4	20	15.8	10.05	.2196
*Podocarpus oleifolius*	4	20	15.4	9.48	.206
*Scyphostelma rugosa*	4	20	15.9	10.1	.2212
*Smallanthus pyramidalis*	4	20	15.5	9.59	.2158
*Stelis sp7*	4	20	15.8	10.05	.2196
*Stelis sp8*	4	20	15.5	9.59	.2158
*Stelis sp9*	4	20	15.5	9.59	.2158
*Weinmannia fagaroides*	4	20	15.4	9.48	.206
*Epidendrum sp1*	4	19.2	17.6	10.49	.2639
*Epidendrum sp2*	4	17.9	17.1	10.65	.4261
*Rubus ulmifolius*	4	17.8	17.2	11.36	.4041
*Baccharis macrantha*	4	17.1	17.6	11.73	.4161
*Rhynchospora macrochaeta*	4	16.9	21.2	11.63	.5953
*Tillandsia biflora*	4	15.2	16.8	11.44	.3915
*Rubus floribundus*	4	14.4	17.2	11.67	.4759
*Oxalis tuberosa*	4	12.8	17.2	11.37	.4971
*Ageratina asclepiadea*	5	81.6	31.1	13.06	.0018
*Ulex europaeus*	5	48.1	20	12.49	.022
*Tillandsia sp 1*	5	47.6	34.7	11.25	.1376
*Vallea stipularis*	5	47.1	30	11.14	.1014
*Bidens rubifolia*	5	46	25.6	12.5	.062
*Miconia ligustrina*	5	45.3	25.2	12.33	.072
*Myrcianthes leucoxyla*	5	42.8	22.8	12.37	.0496
*Baccharis bogotensis*	5	41.6	31.4	11.48	.1598
*Psychotria boqueronensis*	5	40.5	24.7	13.01	.1062
*Monochaetum myrtoideum*	5	34.9	26.3	11.44	.1796
*Ageratina tinifolia*	5	33.3	17.3	11.63	.1168
*Malaxis sp1*	5	33.3	17.9	10.62	.1054
*Peperomia arthurii*	5	30.2	21.2	12.98	.2036
*Myrsine coriacea*	5	30.1	26.3	11.59	.2907
*Weinmannia tomentosa*	5	28.4	25.5	13.01	.3221
*Cavendishia bracteata*	5	28.1	19.3	12.05	.1768
*Morella parvifolia*	5	26.9	18.6	11.95	.23
*Pteridium aquilinum*	5	26.7	19.2	11.47	.2006
*Gaiadendron punctatum*	5	24.3	24.9	13.19	.4045
*Macleania rupestris*	5	23.1	27	14.39	.5399
*Alnus acuminata*	5	16.7	16	10.11	.4161
*Asteraceae sp1*	5	16.7	15.6	9.6	.4119
*Cortaderia nitida*	5	16.7	15.6	9.85	.4005
*Diplostephium rosmarinifolius*	5	16.7	15.7	9.83	.4061
*Elaphoglossum sp1*	5	16.7	16	10.11	.4161
*Epidendrum sisgaense*	5	16.7	15.5	9.44	.4015
*Gaultheria erecta*	5	16.7	15.6	9.85	.4005
*Lantana camara*	5	16.7	15.6	9.6	.4119
*Passiflora capsularis*	5	16.7	15.6	9.6	.4119
*Peperomia angularis*	5	16.7	15.6	9.6	.4119
*Polystichum lehmannii*	5	16.7	15.4	9.42	.3975
*Stachys arvensis*	5	16.7	15.6	9.6	.4119
*Tillandsia sp7*	5	16.7	15.6	9.6	.4119
*Tillandsia sp8*	5	16.7	15.6	9.6	.4119
*Viburnum triphyllum*	5	16	25.4	10.86	.8464
*Tillandsia elongata*	5	14.7	17.1	11.33	.5341
*Thelypteris rudis*	5	14.6	17.3	11.19	.5423
*Rhynchospora nervosa*	5	13.6	16.9	11.4	.5347
*Gaultheria anastomosans*	5	12.8	17	11.42	.5839
*Cranichis sp1*	5	12.5	16.6	11.45	.5659
*Pernettya prostrata*	5	11.8	18.8	12.09	.6569
*Pecluma paradiseae*	5	11.4	16.8	11.24	.6415
*Cardamine ovata*	5	11	18.3	11.87	.6567
*Chromolaena sp2*	5	10.1	16.9	11.31	.7427
*Myrsine guianensis*	5	7.7	16.7	11.21	.9112

## APPENDIX A6

### NMDS tree layer graphs and analysis of variance boxplots

NMDS graphs of the tree layer. (a) Ordination graph of plots in tree species space for axis 1–2: cluster analysis groups are outlined. (b) Ordination graph of plots in tree species space with plotted variables for axis 1–2, with only the variables with ‘p.max = 0.05’ plotted.

The variables that most correlated with the ordination axes (*R*_Sq_ > 0.35 for any of the 3 ordination axis, Table 1) and therefore with species composition and abundances are depicted clockwise. For full variable names and acronyms please refer to Appendix A2
.

Boxplot of analysis of variance for tree layer groups variables. Only variables that significantly differed among various tree groups (Figure 2) are shown. From right to left: age; presence of cattle inside the plot; (c) presence of cattle within 50 m from the plot; presence of cultivated fields within 100 m from the plot; elevation; Shannon's landscape diversity in 1 km buffer; Like adjacencies in 1 km buffer; logging; mean annual temperature; relative humidity.

## APPENDIX A7

### NMDS variables correlation with ordination axes for tree and understory layer

TREES

**Table:**

Variable	NMDS1	NMDS2	*R*_sq_	*p*
elev	0.986936	−0.16111	0.842327	.001
rel_hum	0.557264	−0.83034	0.814799	.001
like_adjacencies	−0.77575	0.631046	0.715877	.001
splitting_index	0.647679	−0.76191	0.712051	.001
patch_cohesion_index	−0.61603	0.787721	0.681776	.001
logg	0.516505	−0.85628	0.633668	.001
greatest_patch	−0.54231	0.84018	0.62502	.001
largest_patch_index	−0.54197	0.840398	0.624739	.001
land_cover	−0.53243	0.846474	0.612971	.001
landscape_porportion	−0.53186	0.846834	0.612778	.001
overall_core	−0.64958	0.760295	0.590216	.001
mean_T	−0.5827	0.812684	0.538388	.001
landscape_shannon	0.392306	−0.91983	0.53266	.001
effective_meshsize	−0.47047	0.882414	0.519565	.002
landscape_division	0.470396	−0.88246	0.519501	.002
%n_CON_ind_T	0.598542	0.801091	0.498647	.001
cult_100	0.828407	−0.56013	0.489726	.001
cattle	0.69714	−0.71693	0.487354	.001
n_CON_ind_T	0.728414	0.685137	0.474397	.001
landscape_simpson	0.333984	−0.94258	0.450877	.003
mliqC	0.917959	0.396676	0.430102	.001
cattle_100	0.535527	−0.84452	0.421884	.003
age	0.230218	0.973139	0.407116	.004
cattle_50m	0.706548	−0.70766	0.387378	.006
road_dist	0.908312	0.418293	0.359839	.001
landscape_pielou	0.549647	−0.8354	0.353878	.004
edge_density	0.922612	0.385729	0.343423	.002
n_patches	0.715658	−0.69845	0.343347	.004
patch_density	0.716517	−0.69757	0.342699	.004
edge_lenght	0.922698	0.385524	0.341936	.002
m_DBH	0.523014	0.852324	0.316577	.004
n.10DBH	0.360665	0.932695	0.29713	.007
fragment	−0.68563	0.727951	0.263566	.016
stems_tree	−0.84893	−0.5285	0.261082	.014
edge	0.690298	−0.72352	0.256775	.025
%n_CON_sp_T	0.596982	0.802255	0.254204	.013
people_density_1km	−0.91227	0.409586	0.253024	.015
mmoss	0.908742	0.417359	0.245418	.022
mean_prec	0.925366	0.379075	0.245257	.013
mean_H	0.356376	0.934343	0.223591	.017
m_cov_inv_U	−0.95141	−0.30794	0.217533	.025
nn_distance	0.589863	−0.8075	0.216971	.031
people_density_5km	−0.92481	0.380421	0.209615	.032
n_inv_sp_U	−0.6223	−0.78278	0.20597	.027
Giniwe	−0.92763	−0.37349	0.20516	.032
mcobT	0.216899	0.976194	0.204958	.028
TPD	−0.78427	−0.62042	0.201613	.044
X._all	0.468041	−0.88371	0.200479	.041
path_dist	0.685608	−0.72797	0.195792	.048
protected	0.615398	0.788216	0.195422	.04
mean_patch	−0.06022	0.998185	0.194229	.042
Giniun	−0.92716	−0.37466	0.185068	.051
HsesMPD	0.988703	−0.14989	0.176882	.068
HsesMNTD	0.348156	−0.93744	0.171425	.065
n_CON_sp_T	0.648282	0.761401	0.17061	.067
cult_50m	0.96786	0.25149	0.16273	.074
n_stems	−0.45592	−0.89002	0.160024	.075
TsesMNTD	−0.81931	−0.57335	0.159924	.081
smallest_patch	0.118311	0.992977	0.156713	.07
median_patch	0.117137	0.993116	0.155408	.075
n_trees	0.219077	−0.97571	0.154968	.096
AGBplot	0.998705	−0.05088	0.154366	.095
cult_500	0.507842	−0.86145	0.153806	.098
mleaf	−0.85047	−0.52603	0.152094	.095
HPD	−0.228	−0.97366	0.150516	.095
TsesPD	−0.51544	−0.85693	0.148752	.102
morqT	0.372927	0.927861	0.145118	.083
mbrioT	−0.13913	0.990274	0.144544	.099
mhelC	−0.99549	0.09491	0.14296	.097
n_FST_ind_T	0.034581	−0.9994	0.141519	.125
H_var	0.642211	0.766528	0.141257	.12
FD	−0.96828	−0.24987	0.140421	.116
mCWD	0.756802	−0.65364	0.137304	.115
FRic	−0.19979	−0.97984	0.137234	.117
HMPD	0.97027	−0.24202	0.129168	.143
TsesMPD	0.417913	−0.90849	0.127914	.142
other	−0.97008	−0.2428	0.126107	.117
mliqT	0.995431	0.095486	0.124934	.171
HsesPD	0.176577	−0.98429	0.121806	.148
mbroT	0.145699	0.989329	0.121332	.176
TsesMNTDABU	−0.99881	0.048778	0.119051	.149
mbroC	−0.26677	0.96376	0.116187	.166
mhelT	−0.16551	0.986208	0.115102	.146
TSR	−0.92753	−0.37375	0.113505	.181
fractal_dimesion_index	0.501947	0.864899	0.110553	.182
m_patchshape_ratio	−0.13742	−0.99051	0.103988	.201
mAGBT	0.649443	0.760411	0.103937	.215
n_sp.10DBH	−0.80905	−0.58774	0.101004	.225
TMPD	0.336675	−0.94162	0.098842	.225
mcobC	0.265827	0.964021	0.097694	.216
sol_rad	−0.17591	0.984406	0.095741	.241
north	−0.40907	−0.9125	0.093814	.22
TPIELOU	0.314002	−0.94942	0.093106	.219
RaoQ	0.764773	−0.6443	0.092153	.264
house_dist	0.316094	0.948728	0.085966	.272
slope	−0.89386	0.448348	0.085597	.29
Hshann	0.169443	−0.98554	0.083351	.282
cattle_500	0.292148	−0.95637	0.080359	.306
n_sp.20DBH	−0.76282	0.646616	0.077075	.32
HSR	−0.34487	−0.93865	0.075683	.323
TMNTD	−0.84452	−0.53553	0.07518	.339
TMPDABU	0.162029	−0.98679	0.074487	.329
Tsimp	0.02132	−0.99977	0.074268	.306
HMNTD	0.7732	−0.63416	0.073592	.32
msoil	−0.6291	0.777325	0.071115	.372
TMNTDABU	−0.99015	−0.14002	0.06572	.38
%_5	−0.20588	−0.97858	0.06549	.365
HsesMNTDABU	−0.52315	−0.85224	0.064366	.377
n.20DBH	0.16129	0.986907	0.060851	.392
Tshann	−0.17924	−0.9838	0.060737	.393
n_FST_sp_T	−0.25137	−0.96789	0.058602	.413
mundstr	−0.55902	−0.82915	0.056845	.435
HPIELOU	0.440781	−0.89761	0.056559	.413
FEve	0.222091	0.975026	0.052515	.481
east	−0.59224	−0.80576	0.051569	.482
Hsimp	0.302214	−0.95324	0.049729	.482
FDis	0.420161	−0.90745	0.046219	.516
DBH_var	0.953164	0.302455	0.043325	.537
n_inv_sp_T	−0.17768	0.984088	0.038882	.571
HsesMPDABU	0.262408	0.964957	0.038792	.567
max_H	0.943325	0.33187	0.038476	.527
track_dist	0.613653	0.789576	0.036408	.575
FDiv	0.063578	−0.99798	0.031273	.634
TsesMPDABU	0.772151	−0.63544	0.029198	.665
morqC	−0.22709	0.973873	0.027614	.666
m_H_understory	0.719951	0.694025	0.018554	.754
HMPDABU	0.997157	0.075357	0.012729	.835
tour	0.074111	0.99725	0.011967	.849
m_cov_nat_U	−0.04515	−0.99898	0.011738	.837
HMNTDABU	−0.99539	−0.09595	0.011378	.848
mbrioC	0.532531	0.84641	0.009829	.886
n_large_trees	−0.57682	0.816872	0.003727	.948

UNDERSTORY

**Table:**

Variable	NMDS1	NMDS2	*R*_sq_	*p*
elev	0.9541	−0.2994	0.7424	.001
%n_CON_ind_T	0.6972	0.7169	0.7030	.001
mliqC	0.8629	−0.5054	0.6210	.001
fragment	−0.4236	0.9058	0.6192	.001
n_CON_ind_T	0.7564	0.6542	0.5868	.001
overall_core	−0.5046	0.8633	0.5779	.001
nn_distance	0.2311	−0.9729	0.5678	.001
n_CON_sp_T	0.4123	0.9110	0.5652	.001
%n_CON_sp_T	0.4612	0.8873	0.5638	.001
road_dist	0.8779	−0.4788	0.5522	.001
edge_density	0.7744	−0.6327	0.5467	.001
edge_lenght	0.7760	−0.6307	0.5458	.001
m_DBH	0.5210	0.8536	0.5361	.001
like_adjacencies	−0.7263	0.6873	0.5287	.001
landscape_pielou	0.3249	−0.9457	0.5282	.001
people_density_1km	−0.6546	0.7560	0.4927	.001
landscape_simpson	0.1586	−0.9873	0.4885	.001
mAGBT	0.5001	0.8659	0.4860	.001
people_density_5km	−0.6587	0.7524	0.4508	.001
effective_meshsize	−0.3264	0.9452	0.4345	.001
landscape_division	0.3269	−0.9450	0.4341	.001
landscape_shannon	0.2587	−0.9660	0.4300	.001
n_stems	−0.3517	−0.9361	0.4254	.001
land_cover	−0.4475	0.8943	0.4112	.002
landscape_porportion	−0.4483	0.8939	0.4105	.002
n_trees	−0.0938	−0.9956	0.4001	.001
cult_500	0.2316	−0.9728	0.3953	.001
mean_H	0.6510	0.7591	0.3926	.001
n.20DBH	0.3331	0.9429	0.3919	.001
greatest_patch	−0.4765	0.8792	0.3789	.003
largest_patch_index	−0.4773	0.8787	0.3783	.003
H_var	0.7528	0.6583	0.3781	.002
mean_patch	0.1234	0.9924	0.3757	.001
age	0.5183	0.8552	0.3631	.001
cattle_100	0.4406	−0.8977	0.3629	.002
n.10DBH	0.5471	0.8371	0.3616	.002
DBH_var	0.3904	0.9206	0.3529	.001
n_patches	0.5521	−0.8338	0.3414	.002
m_patchshape_ratio	−0.2250	−0.9744	0.3411	.001
patch_density	0.5518	−0.8340	0.3409	.002
TsesMNTD	−0.6189	0.7855	0.3379	.004
fractal_dimesion_index	0.3886	0.9214	0.3230	.001
AGBplot	0.6484	0.7613	0.3213	.002
stems_tree	−0.5974	−0.8020	0.3089	.006
mbroT	0.3658	0.9307	0.3069	.005
TsesMNTDABU	−0.4415	0.8973	0.3031	.009
mbroC	0.1503	0.9886	0.3008	.007
median_patch	0.2472	0.9690	0.2962	.002
smallest_patch	0.2492	0.9685	0.2959	.003
TMNTDABU	−0.3654	0.9309	0.2951	.007
n_sp.20DBH	−0.2002	0.9798	0.2940	.003
mmoss	0.7328	−0.6804	0.2935	.011
protected	0.9616	−0.2746	0.2768	.005
mcobT	0.5901	0.8073	0.2766	.009
cattle_50m	0.6646	−0.7472	0.2739	.01
cattle	0.6734	−0.7392	0.2683	.013
HMPD	0.4505	0.8928	0.2675	.013
n_FST_ind_T	−0.0733	−0.9973	0.2597	.017
TsesPD	−0.5789	0.8154	0.2562	.015
HsesMPD	0.5830	0.8125	0.2534	.016
TMPD	0.0440	0.9990	0.2481	.02
morqT	0.7128	0.7014	0.2452	.019
TMNTD	−0.5690	0.8223	0.2441	.017
patch_cohesion_index	−0.7432	0.6691	0.2368	.017
n_large_trees	0.1904	0.9817	0.2271	.019
mleaf	−0.9005	−0.4348	0.2246	.025
mcobC	0.6767	0.7362	0.2211	.031
mliqT	0.7831	−0.6219	0.2199	.026
sol_rad	0.3578	−0.9338	0.2166	.029
tour	0.2983	−0.9545	0.2130	.037
edge	0.6123	−0.7907	0.2109	.033
splitting_index	0.8714	−0.4905	0.2087	.028
cult_100	0.8494	−0.5278	0.2079	.034
n_inv_sp_U	−0.7628	−0.6466	0.2069	.034
TsesMPD	0.0840	0.9965	0.2059	.037
mhelT	0.2106	0.9776	0.1995	.038
max_H	0.7056	0.7086	0.1992	.043
mean_prec	0.8755	−0.4832	0.1907	.053
house_dist	0.5493	−0.8356	0.1902	.055
TMPDABU	0.1551	0.9879	0.1872	.049
TsesMPDABU	0.1618	0.9868	0.1862	.046
Giniwe	−0.5636	−0.8260	0.1815	.049
n_sp.10DBH	−0.6307	0.7761	0.1762	.061
mhelC	−0.4689	0.8833	0.1745	.06
TPD	−0.6826	0.7308	0.1737	.083
cattle_500	0.0966	−0.9953	0.1703	.068
morqC	0.3163	0.9487	0.1693	.069
mean_T	−0.8561	0.5168	0.1561	.075
msoil	−0.2462	0.9692	0.1506	.102
Giniun	−0.5473	−0.8369	0.1506	.096
other	−0.9893	0.1462	0.1492	.078
mundstr	−0.4332	0.9013	0.1469	.108
HMNTD	0.1854	−0.9827	0.1441	.117
m_cov_inv_U	−0.9990	0.0445	0.1427	.124
FDiv	−0.1388	0.9903	0.1402	.122
%_all	0.3145	−0.9492	0.1391	.124
cult_50m	0.7689	−0.6394	0.1348	.101
HsesMNTDABU	−0.8341	−0.5516	0.1219	.157
HPIELOU	0.1081	−0.9941	0.1217	.143
rel_hum	0.8988	−0.4384	0.1211	.152
logg	0.6668	−0.7452	0.1135	.157
m_cov_nat_U	−0.1436	0.9896	0.1131	.167
mCWD	0.6105	0.7920	0.1075	.171
%_5	−0.3215	−0.9469	0.1066	.196
east	−0.8527	−0.5223	0.1063	.162
HsesMNTD	−0.0559	−0.9984	0.1007	.216
HsesMPDABU	0.0924	0.9957	0.0993	.197
TPIELOU	0.4560	0.8900	0.0941	.242
n_FST_sp_T	−0.2760	−0.9611	0.0928	.267
RaoQ	0.8038	0.5949	0.0841	.288
slope	−0.9069	−0.4214	0.0770	.335
FD	−0.7789	0.6272	0.0705	.365
Tshann	0.0868	0.9962	0.0681	.372
Tsimp	0.2034	0.9791	0.0680	.385
track_dist	0.9412	0.3377	0.0674	.375
HPD	−0.8959	0.4443	0.0578	.428
HMNTDABU	−0.7611	−0.6487	0.0570	.42
Hshann	−0.1013	−0.9949	0.0562	.434
FEve	0.4481	0.8940	0.0544	.433
TSR	−0.7679	0.6406	0.0532	.46
mbrioT	0.0471	0.9989	0.0521	.44
m_H_understory	0.2972	0.9548	0.0512	.47
FDis	0.4714	0.8819	0.0497	.486
path_dist	0.7276	0.6860	0.0390	.572
Hsimp	0.0455	−0.9990	0.0381	.57
n_inv_sp_T	0.2033	−0.9791	0.0380	.575
HSR	−0.7537	0.6573	0.0378	.568
north	−0.7210	−0.6929	0.0376	.566
FRic	−0.7253	0.6885	0.0244	.722
HsesPD	−0.4330	−0.9014	0.0243	.722
mbrioC	0.7545	0.6563	0.0112	.868
HMPDABU	0.3434	0.9392	0.0067	.91

## APPENDIX A8

### NMDS understory layer graphs and analysis of variance boxplots

NMDS of understory. (a) ordination graph of plots in understory species space for axis 1–2: no group could be visually distinguished and cluster analysis groups are outlined (b) Ordination graph of plots in understory species space with plotted variables for axis 1–2, with only the variables with ‘p.max = 0.05’ plotted. A table offering variable correlations with the ordination axes is available in Appendix A8.

Boxplot of analysis of variance for understory groups variables. Only variables that significantly differed among various understory groups are shown: elevation; edge density in 1 km buffer; distances from roads; presence of cultivated fields within 100 m from the plot; mean tree AGB; Shannon's landscape diversity in 1 km buffer; people population density in 5 km buffer; presence of cultivated fields within 500 m from the plot.
